# Integrated multi-scale aeromagnetic, gravity, and remote-sensing analysis for mapping basement fabric and structural architecture in the ِِِAswan region, Southern Egypt

**DOI:** 10.1038/s41598-026-56976-7

**Published:** 2026-06-13

**Authors:** Mohamed Khalifa, Mahmoud Ahmed Abbas, Mohammed Atef Mohammed, Barbara Theilen-Willige, Svetlana Kováčiková, Ibrahim Othman, Mahmoud S. Sharkawy

**Affiliations:** 1 National Research Institute of Astronomy and Geophysics, Helwan, Cairo, 11421 Egypt; 2 Department of Earth, Environmental and Resources Sciences (DiSTAR), University of Naples Federico II, Naples, 80126 Italy; 3 Geology Department, Faculty of Science, Qena University, Qena, 83523 Egypt; 4 Department of Geology, Faculty of Science, Sohag University, Sohag, Egypt; 5 Retired from TU Berlin, Institute of Applied Geosciences, Waldstrasse 11a, 23683 Scharbeutz, DE Germany; 6 Institute of Geophysics, Czech Academy of Sciences, Boční II/1401, Praha 4, Prague, 14100 Czech Republic; 7 Geology Department, Faculty of Science, Tanta University, Tanta, 31527 Egypt; 8 Department of Urban Management, Graduate School of Engineering, Kyoto University, Kyoto, 615-8540 Japan; 9 Department of Petroleum Geology, Faculty of Petroleum and Mining Sciences, Matrouh University, Marsa Matrouh 51511, Matrouh, Egypt

**Keywords:** Aeromagnetic data, Bouguer gravity, Depth to basement, Structural lineaments, Remote sensing, DEM hillshade, Euler deconvolution, Aswan, Egypt, Environmental sciences, Natural hazards, Solid Earth sciences

## Abstract

An integrated imaging workflow combining aeromagnetic, gravity, optical, and radar satellite datasets was applied to characterize basement structures beneath the Aswan area, southern Egypt. Optical and radar satellite data lineament analyses, together with Digital Elevation Model (DEM) derived hillshades, and automated lineament extraction revealed a dominant NW–SE orientation related to the Pan‑African shear fabric with minor E–W unloading joints, and reactivated NE–SW shear trends. Bouguer gravity anomalies (–48.3 to − 13.5 mGal) clustered spatially and are characterized by higher values (–13.5 to − 22.0 mGal) near Aswan, aligned with dense crystalline horsts, whereas lower anomalies (–35.5 to − 48.3 mGal) delineated sediment-filled grabens, dominant along the west and the southeast. High-pass filtering highlights shallow N-S lineaments parallel to the Nile valley and NE-SW fractures. In contrast, low-pass filtering mapped the broader basement geometry, showing a gentle NW-SE to E-W slope with uplifted shoulders east of the study area. Two-dimensional magnetic modeling, supported by Euler deconvolution, revealed significant basement depth variation across the study area, ranging from about 350–400 m in shallow zones to 1,800–2,600 m in deeper sectors. These integrated imaging results revealed horst–graben structures, identified favorable targets for groundwater and mineral exploration, guided infrastructure planning, and demonstrated the value of integrated geophysical workflows.

## Introduction

**Fig. 1:**
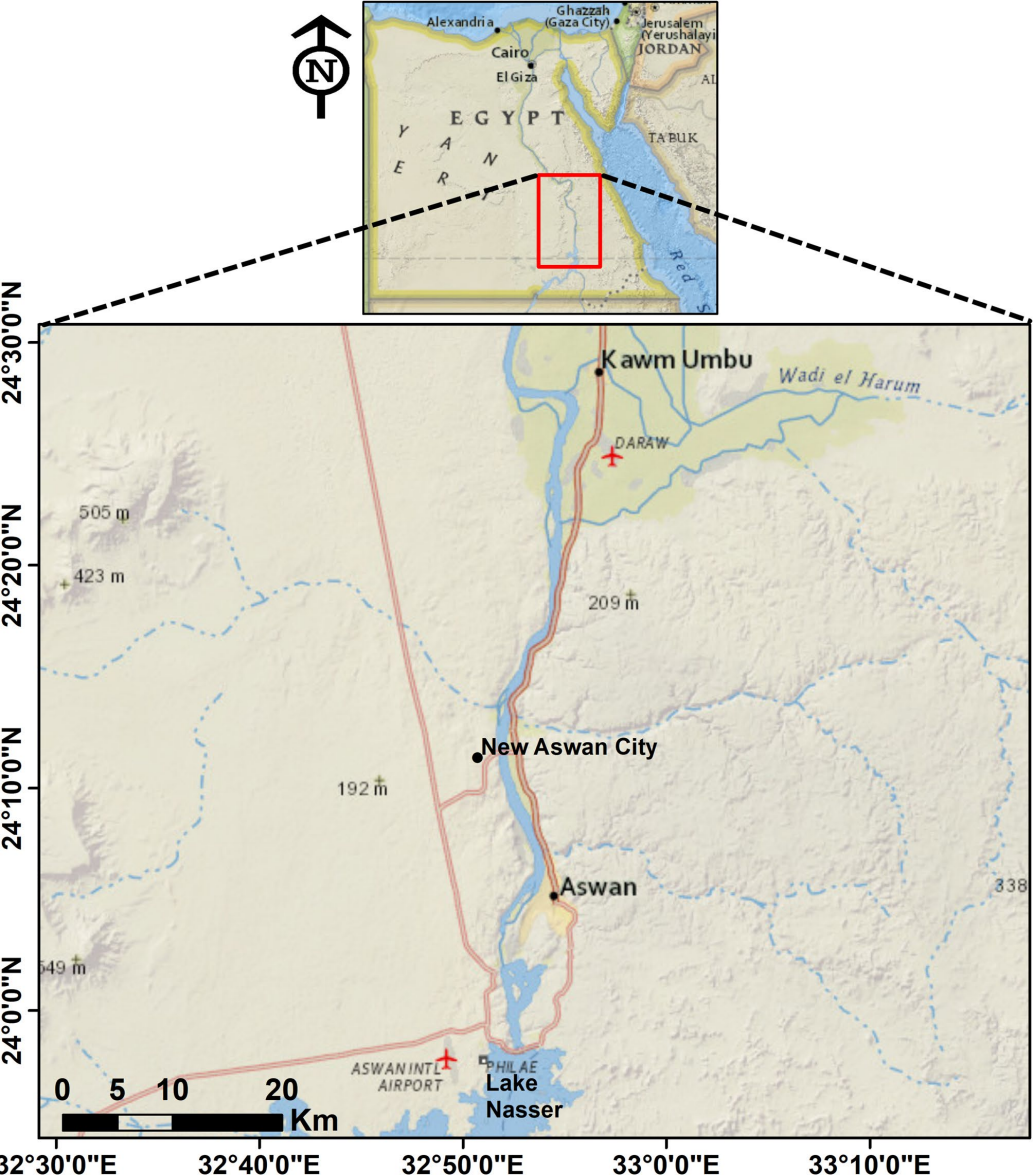
Location map of the study area. Map created using ArcGIS Pro (version 3.7; https://www.esri.com/en-us/arcgis/products/arcgis-pro/overview).

The rugged, arid desert terrain poses major challenges for geological surveys, which involve extreme climatic conditions, complex relief, and difficult logistics that often limit detailed ground surveys and leave significant gaps in high-resolution geological, structural, and mineralogical data. Advances in multisensory satellite remote sensing (Landsat, Sentinel, ALOS PALSAR), combined with geophysical potential field datasets, proved effective in overcoming these limitations. This has enabled the discrimination of lithology, mapping of hydrothermal alteration zones, and extraction of structural features (faults, folds, etc.), both local and regional joints^1,2^. The application of satellite gravity and magnetic data provided essential constraints to refine the subsurface geology, structural framework, lithological boundaries, and tectonic setting^3,4^. Aeromagnetic data are very effective for imaging fault systems, fold geometry, and lithological contacts, as well as for enhancing the interpretation of regional tectonic patterns^5–7^. The integration of multispectral/radar imagery, DEM-derived hillslopes, and gravity and magnetic data improved the delineation of basement structural and shallow intrusions below the surface cover, reduced interpretation ambiguities, and guided targeted field investigations. This integrated approach improves the accuracy of subsurface structural mapping and enables the identification of deep-seated faults, shear zones, and intrusive bodies that control basin evolution and the distribution of magnetic anomalies. Overall, it helped prioritize structurally favorable subsurface zones that may influence the occurrence and distribution of natural resources, including groundwater and mineral deposits, depending on the geological setting. This reduces exploration costs and associated risks while supporting sustainable development in complex and inaccessible terrain. The study area is located in southeastern Egypt within the Aswan region along the Nile Valley (approx. 24°00′N–24°20′N, 32°40′E–33°10′E) and comprises urban settlements and adjacent desert outcrops. The primary aim of this study is to delineate the structural framework of the Aswan region, southeastern Egypt, through an integrated analysis of reduced to pole (RTP) magnetics, Bouguer gravity, and remote sensing (optical and radar), complemented by Digital Elevation Model (DEM)-based hillshade and automated lineament extraction. The location map and data coverage are presented in Fig. 1.

## GEOLOGY OF THE STUDY AREA

Many researchers have studied the Aswan region’s geology in detail^8–13^. The Precambrian basement rocks are mainly composed of granitic and granodioritic intrusions, together with associated metamorphic units exposed near Lake Nasser, particularly east and south of Aswan^14^.

The oldest sedimentary unit in the region, the Cretaceous Nubian Sandstone, lies unconformably on top of the basement complex. This unit is found in large exposures north of Aswan and dominates the Nubian Plain west of the Nile. The three formations that make up this area are the Um Barmile Formation (Campanian in age), the Timsah Formation (fine clastics and oolitic marine strata of Coniacian–Santonian age), and the Abu Agag Formation (fluvial deposits of likely Turonian age). It is primarily composed of ferruginous sandstones, sandstones, and clays^14^.

**Fig. 2:**
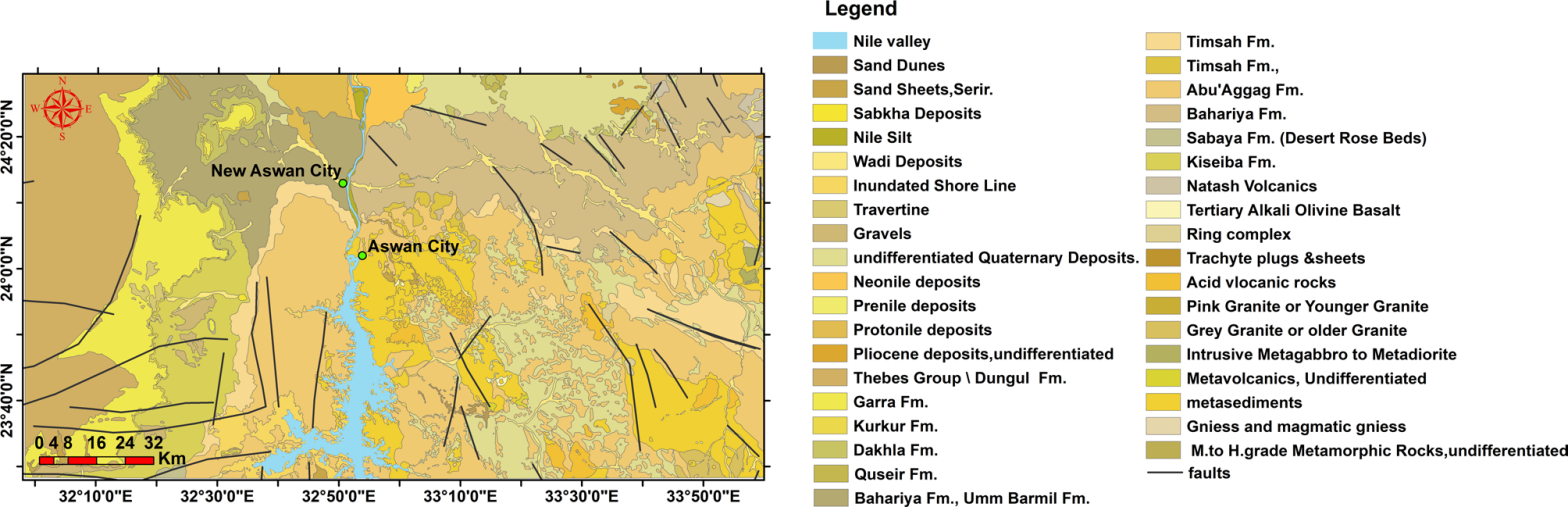
Geological map of the study area. The map was created using ArcGIS Pro 3.7 (https://www.esri.com/en-us/arcgis/products/arcgis-pro/overview).

The well-developed Dakhla Formation shales along the western Limestone plateau represent later Cretaceous deposits. The Kurkur, Garra, and Dungul formations comprise Paleocene-Eocene marine limestones that cap the plateau (Fig. 2). The plateau, which rises to an average elevation of roughly 350 m, forms a noticeable escarpment that borders the Nubian Plain to the west. Its terrain ranges greatly, from wide, level areas to rough, hummocky terrain with resilient rock hills and benches^13^.

Together with these main units, the Aswan region also has a variety of calcareous-evaporitic sediments, with tufa and calcite being especially prevalent near the plateau’s edges. Widespread Quaternary deposits include playa deposits, gravels, conglomerates, alluvium–eluvium, and surficial sediments, with alluvial materials predominating.

The complex structure of the Aswan region reflects the interaction between Phanerozoic deformation and Precambrian basement tectonics. A network of faults and shear zones that primarily trend in NW–SE, E–W, and NNE–SSW directions cut through the basement rocks^9–11^. These structural patterns were later reactivated during subsequent geologic periods and are linked to Precambrian tectonic events associated with the Pan-African orogeny. The basement’s faults frequently served as weak areas, regulating the thickness variations and deposition of the younger sedimentary successions and the Nubian Sandstone that covered it^14^.

In the sedimentary cover, important structural features include fault block systems, dips, and folds that develop in response to both regional tectonics and local basement controls. The NW-SE trending Kalabash fault system is the most prominent structural feature of the region and has been linked to several seismic events in the Aswan region^15–18^. This fault system, together with subducting faults, has a significant impact on the groundwater circulation, surface geomorphology, and stability of the Aswan High Dam reservoir. Overall, the structural fabric of the area demonstrates strong basement control, repeatedly reactivated through geologic time, shaping both the lithological distribution and the hydrogeological framework of the region.

## Materials and methods

### Remote Sensing and GIS data

**Fig. 3:**
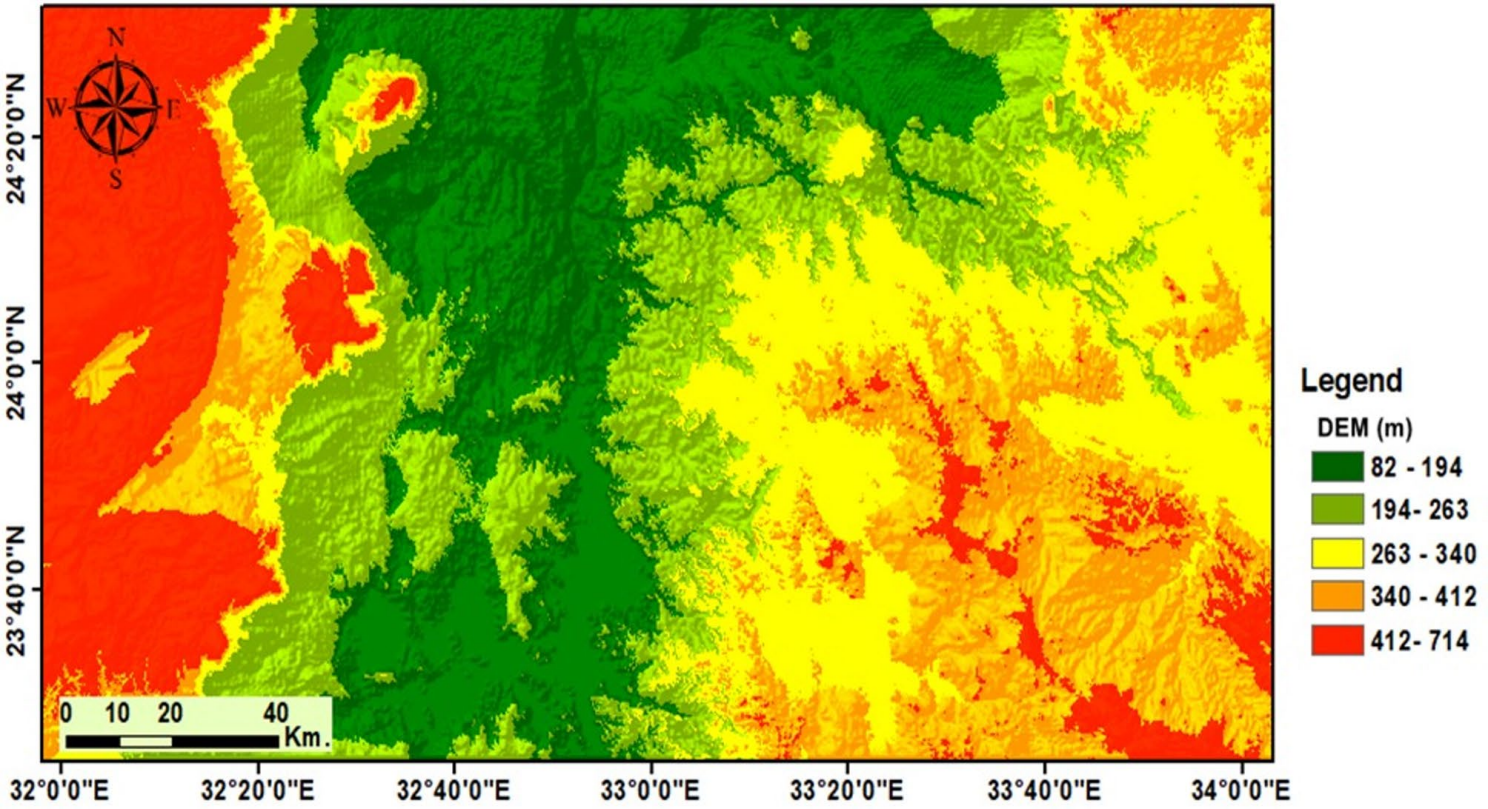
Digital elevation model shows the major topographic features of the study area.

**Fig. 4:**
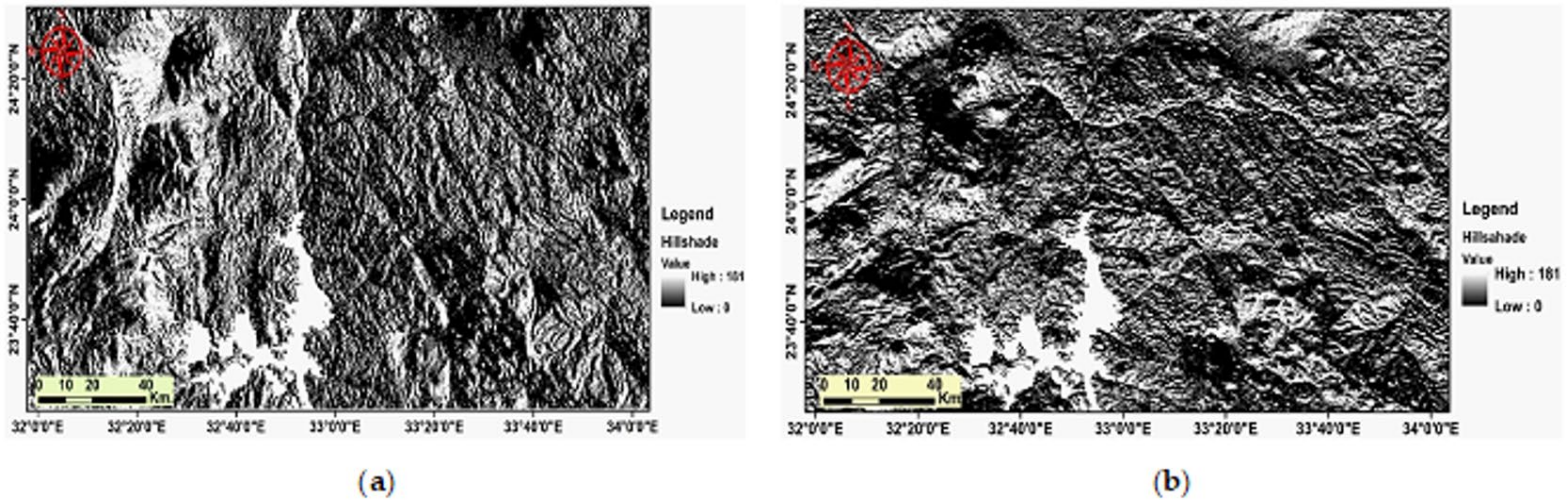
Hillshade map of the study area generated using sun azimuths of (a) 0°, 45°, 90°, and 135°; (b) 180°, 225°, 270°, and 315°.

**Fig. 5:**
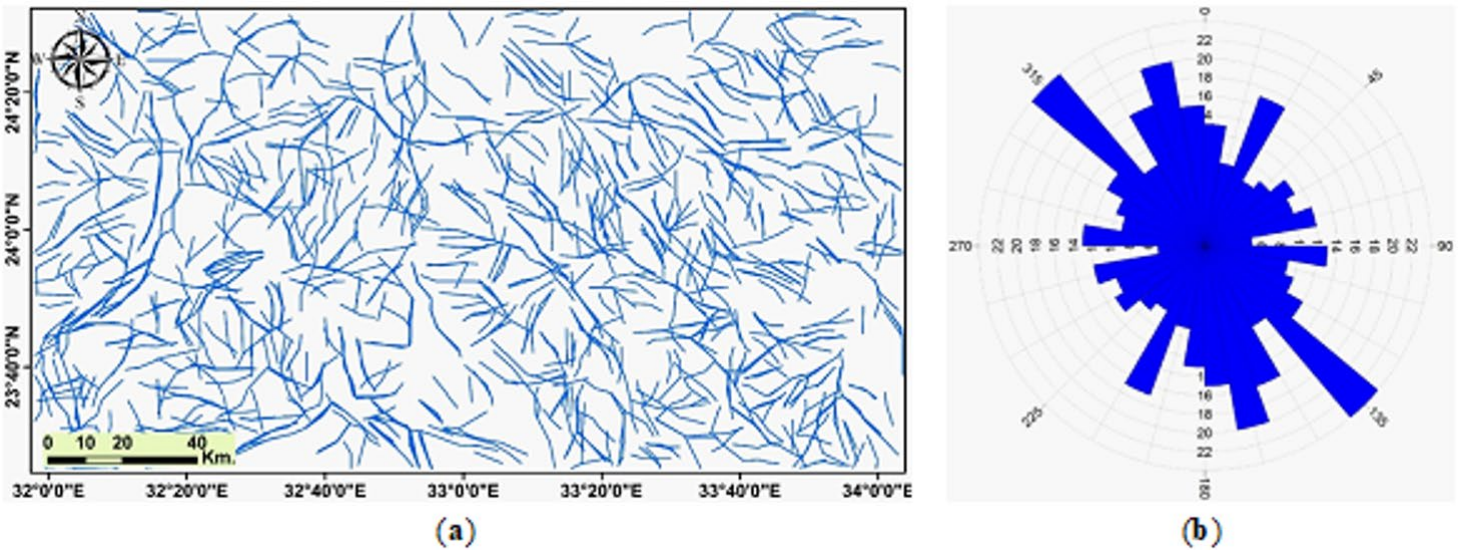
(a) Lineament map of the study area, generated from shaded relief imagery at sun azimuths 0°, 45°, 90°, and 135°; (b) The azimuth‑frequency (rose) diagram.

**Fig. 6:**
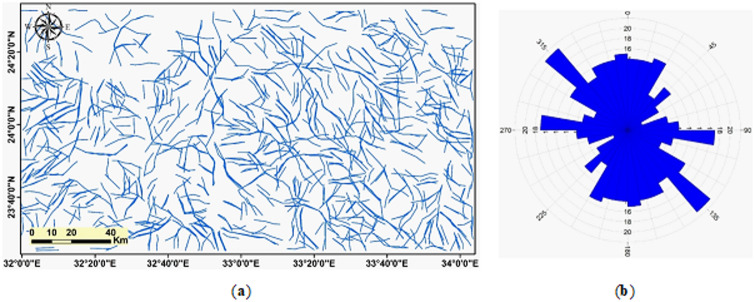
(a) Lineament map of the study area, generated from shaded relief imagery at sun azimuths 180°, 225°, 270°, and 315°; (b) The azimuth‑frequency (rose) diagram.

Based on Landsat 8/9 (provided by the USGS, Earth Explorer, and Sentinel 2, provided by the Copernicus browser/ESA) and ALOS PALSAR/JAXA optical satellite data, and Sentinel 1 radar data/ESA, a visual lineament analysis was carried out. The Advanced Land Observing Satellite (ALOS), Phased Array type L-band Synthetic Aperture Radar (PALSAR) from the Japan Aerospace Exploration Agency (JAXA), provided data with 12.5 m spatial resolution. Digital image processing methods using SNAP /ESA and ENVI software were used to derive image products such as enhanced RGB images or Principal Component (PC) images. Elevation data were extracted from the GMTED2010 tile GMTED2010N10E030, a 7.5-arc-second (~ 250 m) Geo-TIFF dataset provided by the USGS/EROS Center and acquired on 11 November 2010. Digital Terrain Elevation Data (DTED) from the Shuttle Radar Topography Mission (SRTM), Canadian elevation data, Spot 5 Reference 3D data, and data from the Ice, Cloud, and land Elevation Satellite (ICE-Sat) (U.S. Geological Survey (USGS) and the National Geospatial-Intelligence Agency (NGA). The tile spans latitudes 10°–30°N and longitudes 30°–60°E (center: 20°N, 45°E), with elevation values referenced to the EGM96 geoid (accessible via USGS Earth Explorer). GMTED2010 integrates multiple higher-resolution sources into seven statistical raster layers (minimum, maximum, mean, median, standard deviation, systematic subsample, and breakline emphasis). After clipping to the study area, the mean elevation grid was imported and reprojected to WGS 84/UTM Zone 36 N using the “Project Raster” tool (Fig. 3). However, because GMTED2010 has a relatively coarse spatial resolution for local-scale structural mapping, the extraction of very small lineaments, narrow dykes, and subtle surface features may be limited. For this reason, the DEM-based interpretation was used mainly to support regional structural trends rather than to resolve fine-scale features. Hillshades were generated from this grid at eight azimuths (0°, 45°, 90°, 135°, 180°, 225°, 270°, 315°) with a 45° solar altitude using the Spatial Analyst toolbox (Fig. 4a and b). Lineaments were subsequently extracted and analyzed through rose diagram orientation and circular statistics (Figs. 5 and 6).

### Gravity Data

The gravity dataset utilized in this study is based on the Earth Gravitational Model 2008 (EGM2008)^19^, recognized as one of the most comprehensive global representations of the Earth’s gravity field. EGM2008 integrates diverse data sources, including terrestrial gravity measurements supplied by the National Geospatial-Intelligence Agency (NGA), the Bureau Gravimétrique International (BGI), and the U.S. Naval Observatory (USNO). Additionally, it incorporates airborne gravity surveys conducted in areas with sparse ground-based data. Over oceanic regions, gravity anomalies are constrained using satellite altimetry data, while long-wavelength components are derived from observations conducted by the GRACE satellite mission. Enhancing its accuracy, the model uses detailed topographic and bathymetric corrections derived from global digital elevation models such as DTM2006.0 and SRTM, complemented by shipborne bathymetric data.

For the estimation of Bouguer anomalies, a standard crustal density value of 2670 kg/m³ was employed. The resulting gravity grid is composed of a spatial resolution of approximately 5 arc-minutes (~ 9 km). Although EGM2008 provides a robust global representation of the Earth’s gravitational field, its resolution is best suited for regional to intermediate-scale geological and structural interpretations. Within the scope of this study, this resolution is adequate to identify major fault systems, basement structures, and large-scale tectonic trends in the Aswan region. However, it is important to note that this dataset lacks the fine resolution required to detect small-scale or shallow local structures. The absence of complete high-resolution terrestrial gravity measurements across the study area further limits localized analyses. Consequently, the EGM2008-derived Bouguer anomaly dataset represents the most reliable and comprehensive resource available for this regional-scale investigation.

**Fig. 7:**
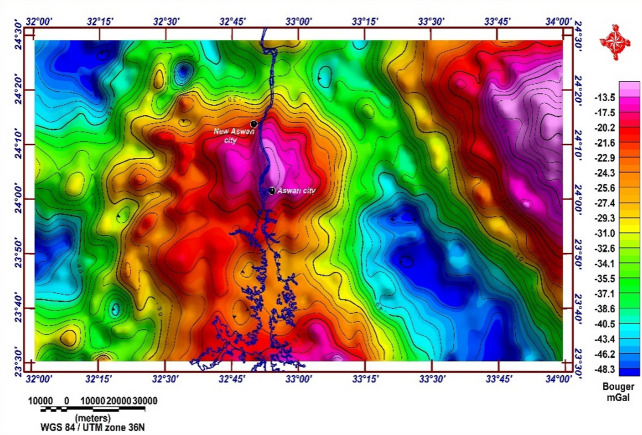
Bouguer gravity map of the Aswan area (mGal).

To support structural interpretation, the Bouguer gravity data were analyzed to separate anomalies based on their wavelengths. The resulting Bouguer gravity map (Fig. 7) showcases distinct patterns of subsurface density variations. Longer-wavelength anomalies are generally indicative of deep-seated crustal features, such as variations in basement topography and crustal thickness. In contrast, shorter-wavelength anomalies are linked to shallower sources, including intrusive bodies, basement elevations, and lateral density changes in sedimentary basins. These findings collectively highlight the effectiveness of the gravity dataset in capturing regional structural trends. Furthermore, the data provide a strong foundation for applying wavelength-based filtering methods and undertaking subsequent geophysical interpretations at varying depths.

### Magnetic Data

The airborne magnetic datasets used in this study were acquired in 1984 through the Minerals, Petroleum, and Groundwater Assessment Program (MPGAP), a cooperative initiative of the Egyptian General Petroleum Corporation (EGPC), the Aero-Service Division (ASD), the Western Geophysical Company of America, and the Egyptian Geological Survey and Mining Authority (EGSMA). Although the survey was conducted several decades ago, the study area has remained untouched, and geophysical data continue to provide a valid representation of the subsurface geology and mineralogical characteristics of the area. The survey used traverse lines oriented towards N 45°E with a nominal distance of 1.0 km, while tie lines, with a distance of approximately 10 km apart, were oriented vertically at N 135°E^20^. Magnetic data were represented by 1: 50,000 scale total magnetic intensity (TMI) and reduced to pole (RTP) aeromagnetic grids.

**Fig. 8:**
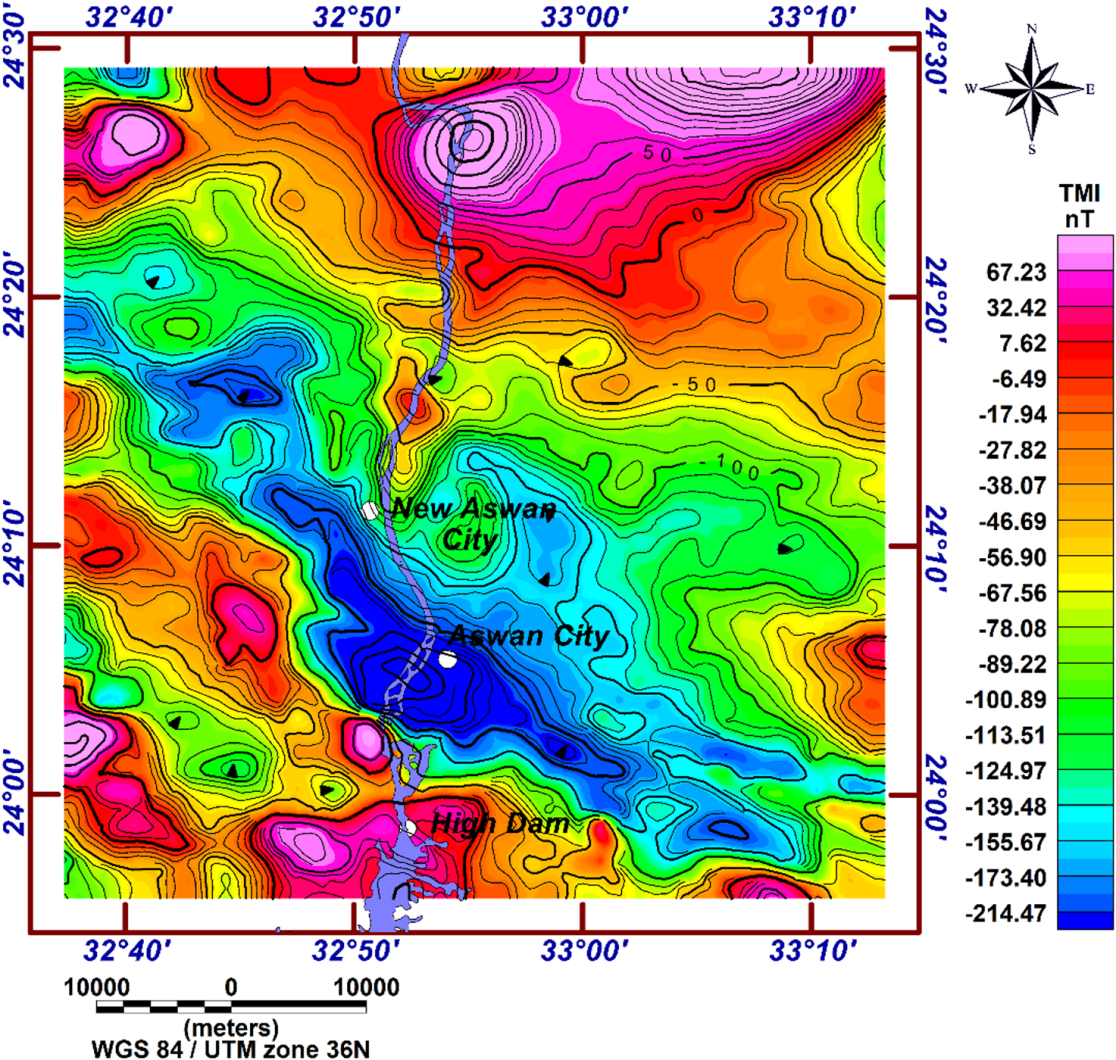
Total field aeromagnetic anomaly map of the Aswan area (nT).

The airborne magnetic data were corrected for the International Geomagnetic Reference Field (IGRF) by the survey contractor to obtain magnetic anomaly values. For presentation and compatibility purposes, a constant value of 42,425 nT was added to convert the anomaly data back to a Total Magnetic Intensity (TMI) representation. The total field magnetic anomaly is shown in Fig. 8 after subtracting the constant value.

The gravity and aeromagnetic datasets used in this study have different spatial extents due to their different acquisition sources. Both datasets were used in their original form, and the integrated interpretation was performed within the defined study area without modifying the original grid boundaries. It should be noted that the airborne magnetic dataset used in this research, obtained in 1984, represents the most comprehensive and available magnetic dataset currently available for the study area. While the 1 km flight-line spacing may limit the resolution of very shallow and small-scale structures, the dataset remains highly suitable for regional to intermediate-scale structural interpretation, including major fault systems, basement relief, and tectonic trends. Conducting new high-resolution ground magnetic surveys over such a wide and geologically complex area would require substantial logistical effort and financial resources. Therefore, the available airborne dataset provides a scientifically valid and practical basis for the present regional-scale investigation. Future studies integrating higher-resolution airborne or ground magnetic data are recommended to further refine shallow structural details.

## DATA PROCESSING AND INTERPRETATION

### Remote Sensing and GIS data

**Fig. 9:**
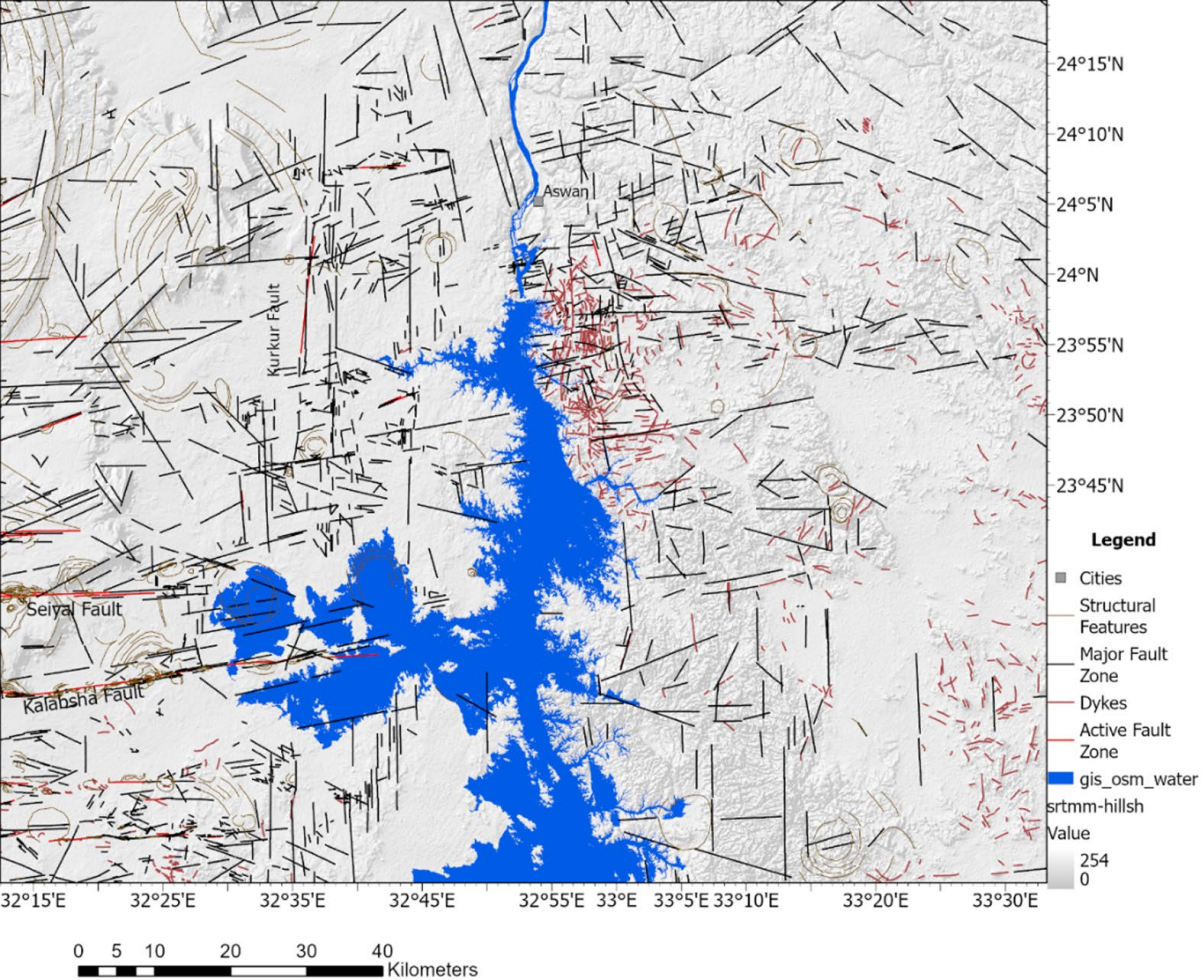
The overall lineament map derived from the interpretation of Landsat and Sentinel-2 imagery. The original Landsat data were downloaded from the US Geological Survey, EarthExplorer (https://earthexplorer.usgs.gov/), and the Sentinel-2 data were downloaded from the European Space Agency (ESA), Copernicus Browser (https://browser.dataspace.copernicus.eu/). The map was created using ArcGIS Pro 3.7 (https://www.esri.com/en-us/arcgis/products/arcgis-pro/overview).

The structural/tectonic knowledge gained from visual and automated lineament analysis and geophysical data is an important input for groundwater and mineral exploration, as it supports the detection of fault and fracture zones that influence groundwater flow or mineralization. The special focus of the evaluations was on mapping dykes and dyke swarms that intruded into zones of weakness, thereby tracing the fault pattern at the time of their intrusion. The result of the visual evaluation of the different satellite images is summarized in Fig. 9.

**Fig. 10:**
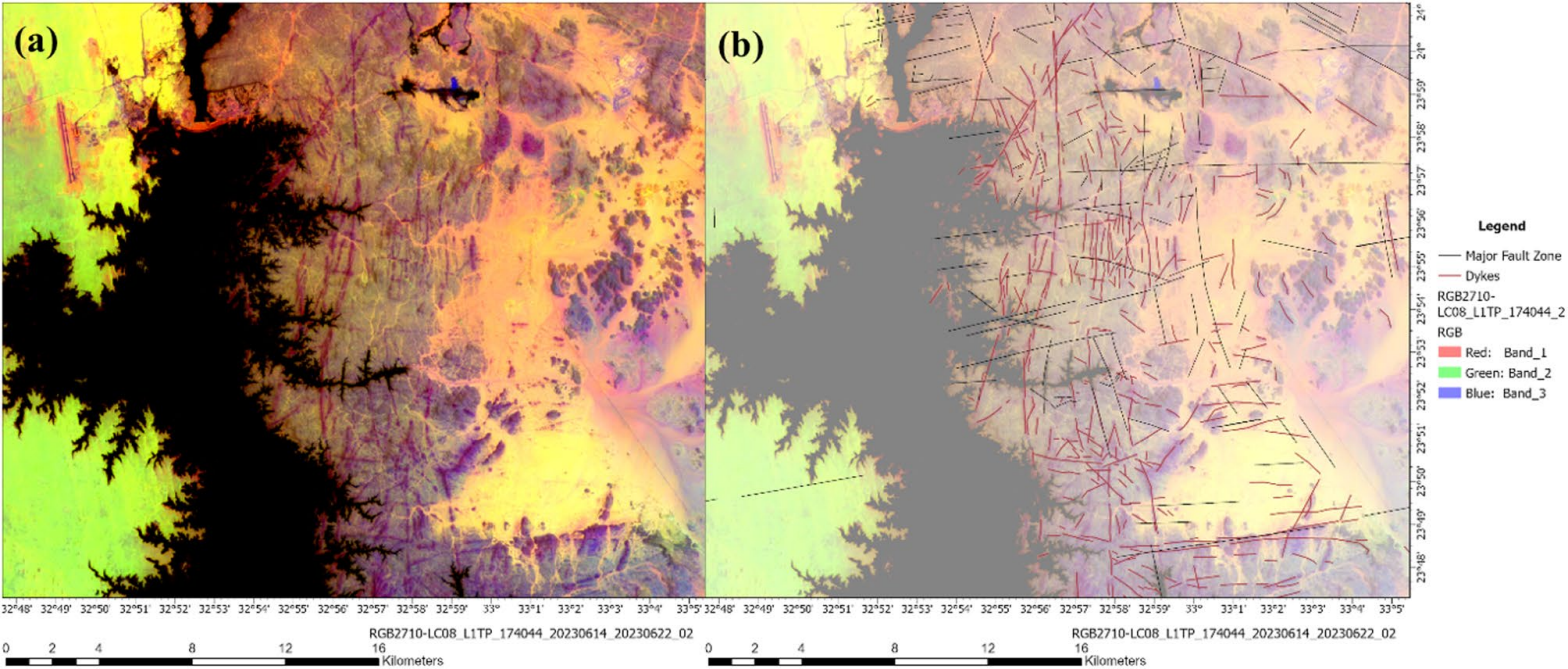
Structural evaluation of a Landsat 9 scene of the Aswan area and corresponding dyke map: (a) True-color RGB composite of the Landsat 9 scene; (b) Extracted lineament analysis highlighting dyke swarms intruded into the granitic basement. The Landsat data were digitally processed using ENVI^®^ (NV5 Geospatial; https://www.nv5geospatialsoftware.com/Products/ENVI) and ArcGIS Pro 3.7 (https://www.esri.com/en-us/arcgis/products/arcgis-pro/overview) for GIS-integrated evaluation.

**Fig. 11:**
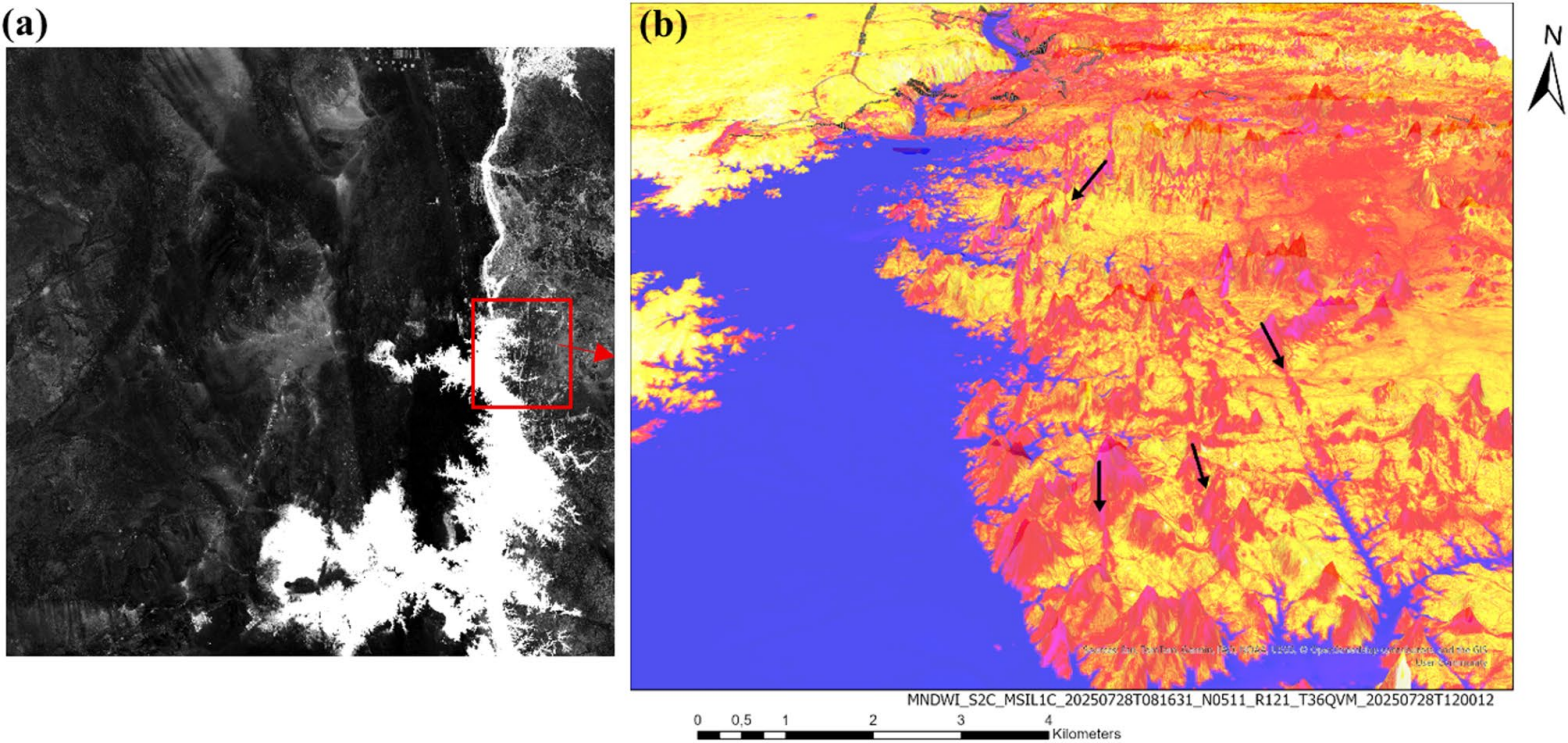
(a) Sentinel 2 derived Modified Normalized Difference Water Index (MNDWI) image. The Modified Normalized Difference Water Index (MNDWI) uses green and SWIR spectral bands for the enhancement of open water features and higher soil moisture. (b) Amplified, color-coded MNDWI scene in a 3D perspective view (with 10 x vertical exaggeration) of the Aswan area, showing an enhanced visibility of sub-vertical dyke intrusions oriented predominantly N–S, E–W, and NE–SW direction (see black arrows). Digital image processing was carried out using SNAP 13 (ESA/STEP; https://step.esa.int/main/) and ArcGIS Pro 3.7 (https://www.esri.com/en-us/arcgis/products/arcgis-pro/overview).

**Fig. 12:**
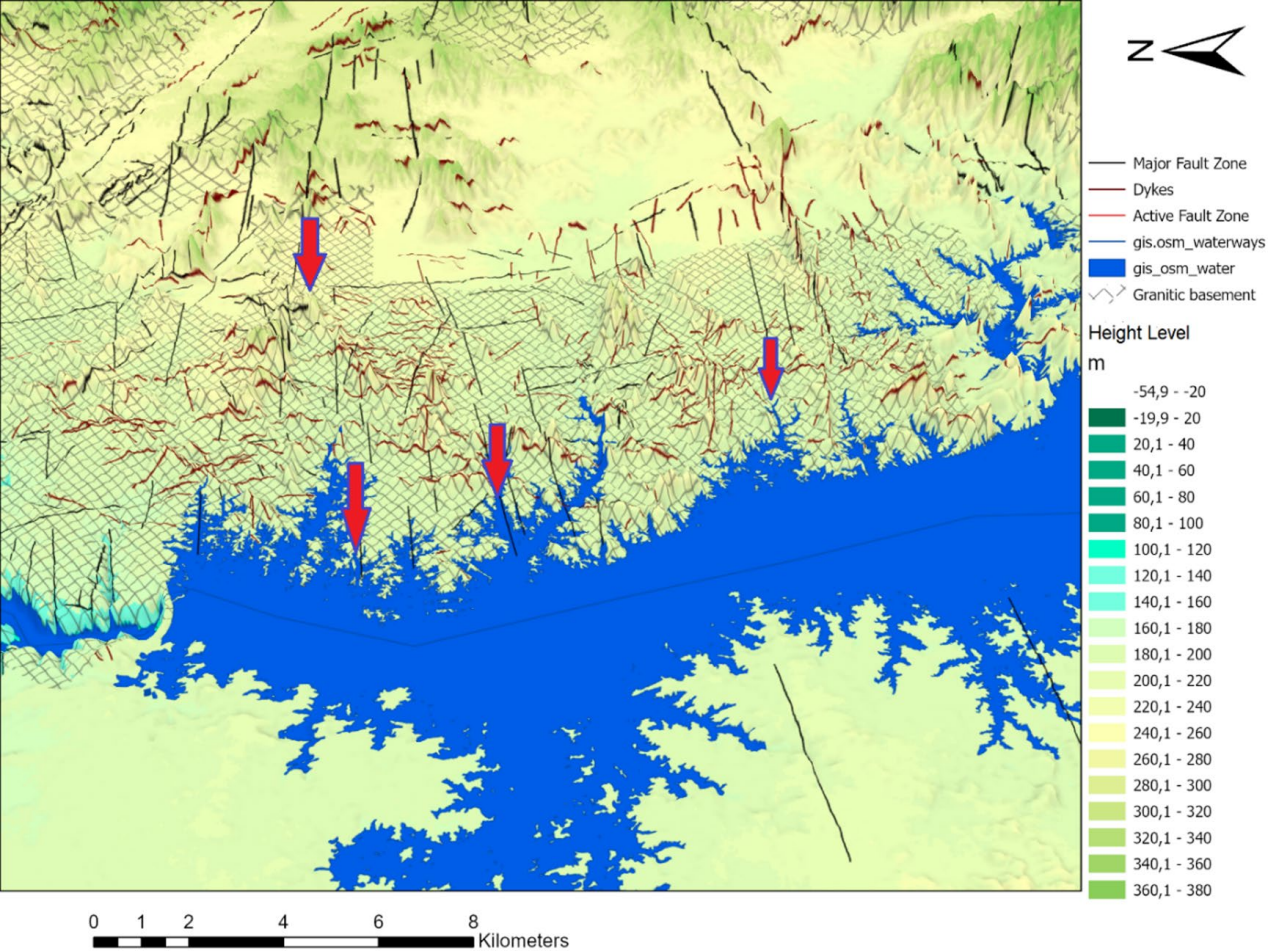
Eastward-looking 3D perspective view highlighting lineaments and dykes (dark-red lines) with key inferred influences on groundwater flow (red arrows) into the reservoir with a SRTM DEM derived height level map in the background. The figure was created using ArcGIS Pro 3.7 with the 3D Analyst extension (https://www.esri.com/en-us/arcgis/products/arcgis-pro/overview).

Faults have a positive effect on groundwater flow, where they enhance permeability through increased fracture density or structural weakening of the granitic basement bedrock. Conversely, faults can disrupt groundwater flow where impermeable fault planes block circulation, redirect flow to other horizons, channel water along the fault plane itself, or force discharge at surface springs^21^. Dyke intrusions of varying age, mineralogical composition, and morphologies were ubiquitous in eastern Egypt. Dykes-subvertical, sheet-like intrusions that typically exploited extensional faults and fracture networks were noted. Although highly weathered dykes sometimes acted as preferred groundwater conduits where they were surrounded by low-permeability host rocks, they more often acted as hydraulic barriers, producing compartmentalized aquifers and stepwise hydraulic head distributions. Groundwater compartments that are irregular in volume, shape, and structure develop in areas affected by dykes^22^. Dyke intrusions are clearly visible on Landsat and Sentinel 2 images as well as on the radar images in the south of the city of Aswan embedded in the granitic basement rocks (Fig. 10), most of them oriented in N-S, E-W, and NE-SW directions. It can be assumed that dykes form more hindrances and barriers for groundwater flow, of course, depending on their weathering degree, whereas surface-near fault and fracture zones form pathways for the flow. The dykes become visible as well on the Sentinel 2-based Modified Normalized Difference Water Index (MNDWI) image product (Fig. 11). Figure 12 shows a perspective view of the Aswan reservoir to visualize those fault zones with arrows. Thus, lineament analysis can support the detection of groundwater flow paths.

### Gravity Data

**Fig. 13:**
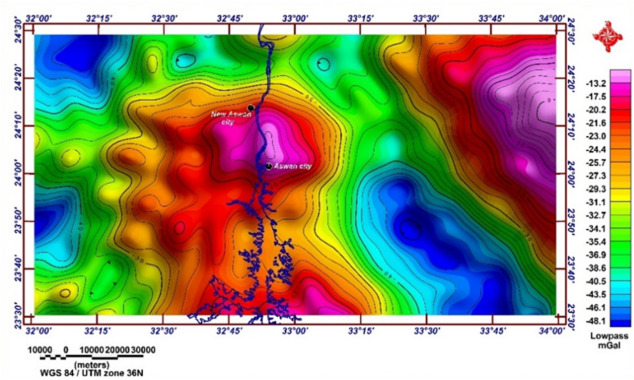
Low-pass gravity filter map of the study area using an effective cutoff wavenumber of 8.090 km⁻¹ (cycles/km).

**Fig. 14:**
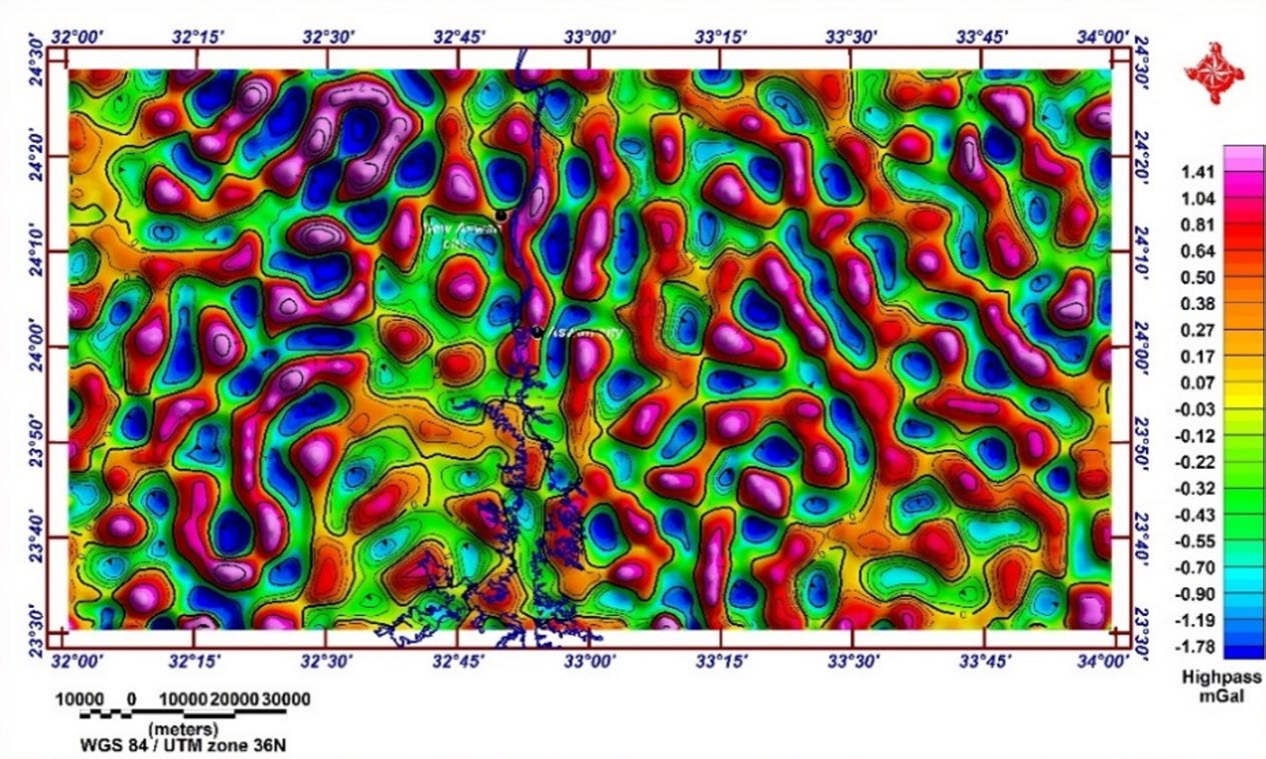
High-pass gravity filter map of the study area using an effective cutoff wavenumber of 8.090 km⁻¹ (cycles/km).

**Fig. 15:**
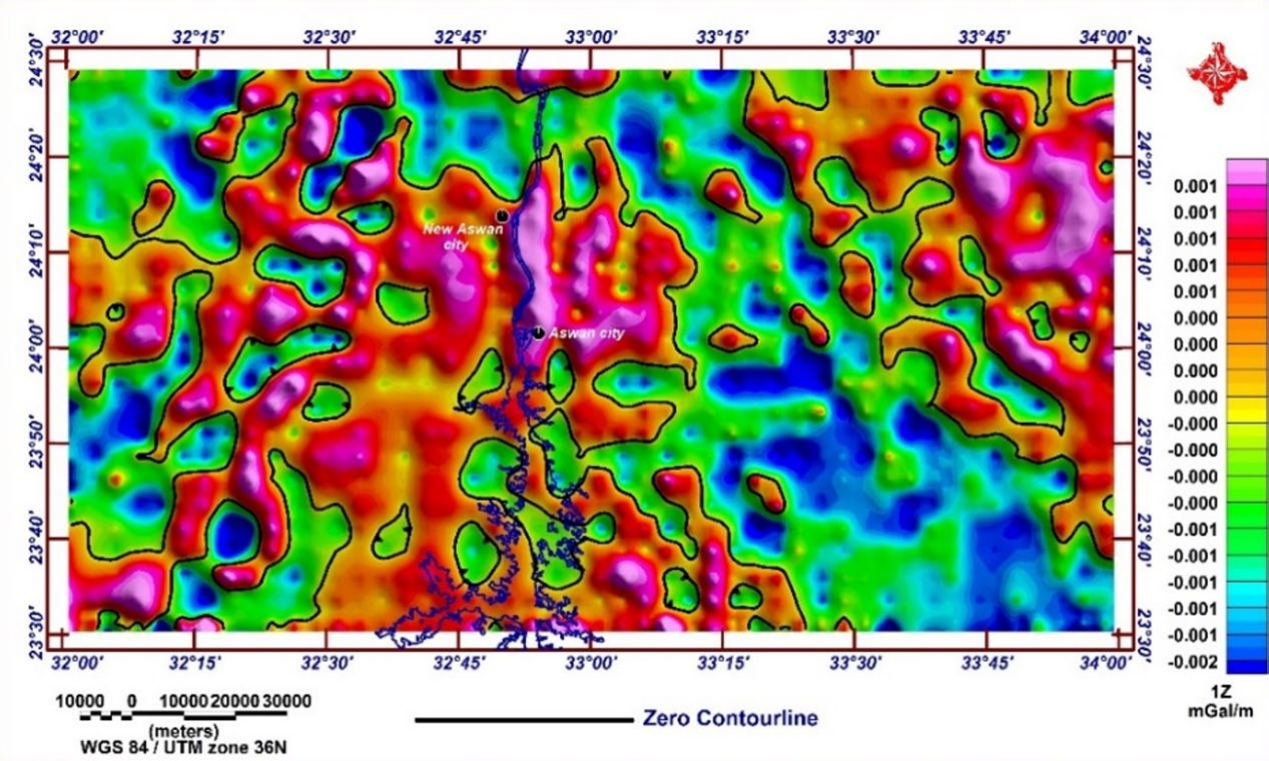
The first vertical derivative (FVD) applied to the Bouguer gravity map improves short-wavelength anomalies associated with near-surface structures.

**Fig. 16:**
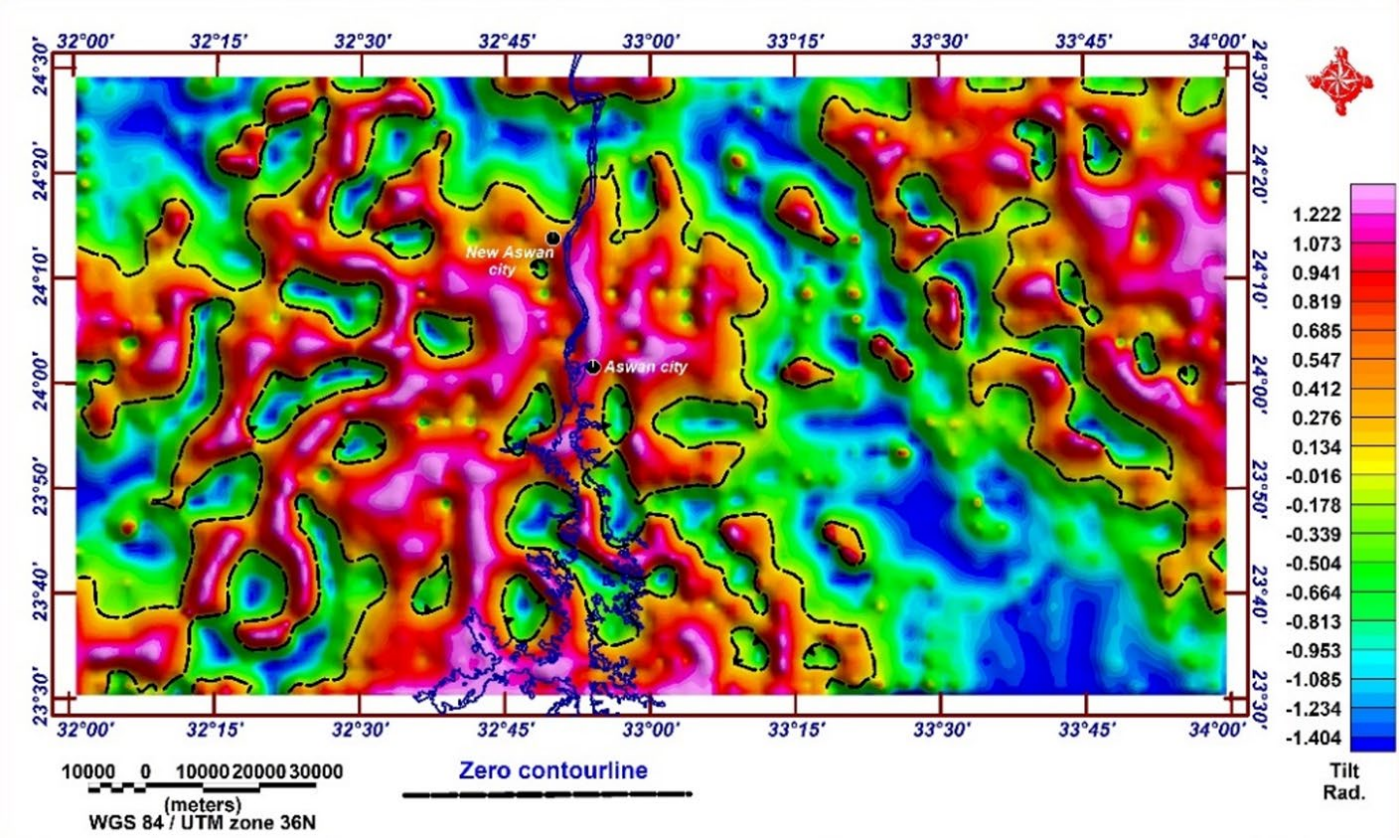
Tilt Derivative filter applied on the Bouguer gravity map of the Study Area.

**Fig. 17:**
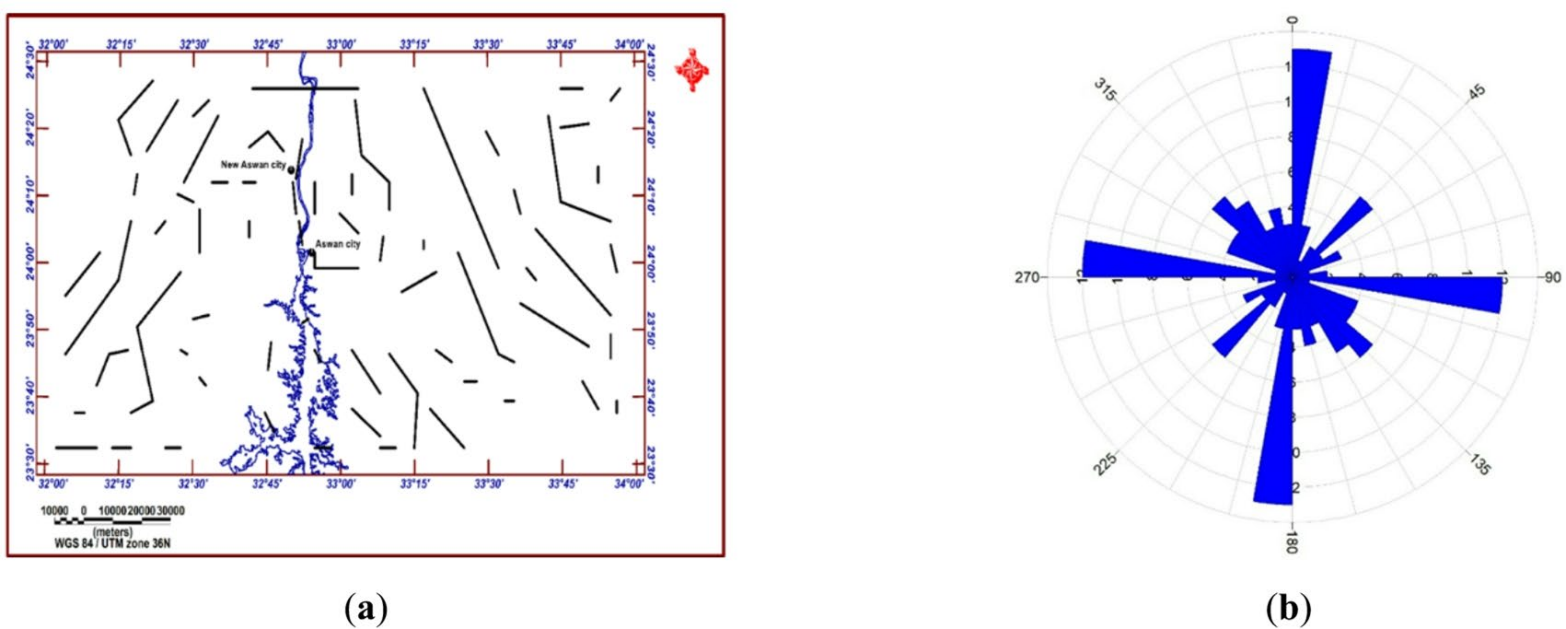
(a) Main structural lineaments deduced from the Bouguer gravity anomaly map; (b) Their azimuth‑frequency (rose) diagram.

**Fig. 18:**
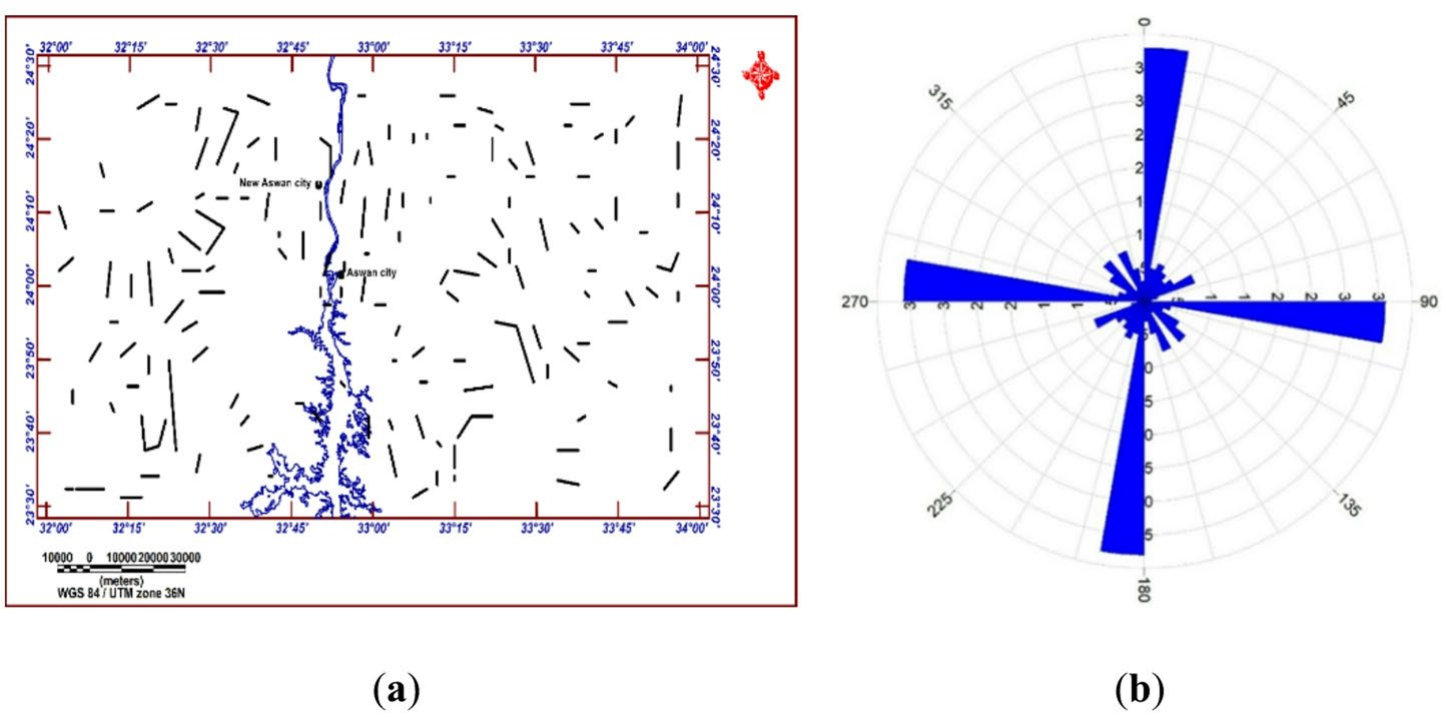
(a) Main structural lineaments deduced from the High-pass gravity anomaly map; (b) Their azimuth‑frequency (rose) diagram.

**Fig. 19:**
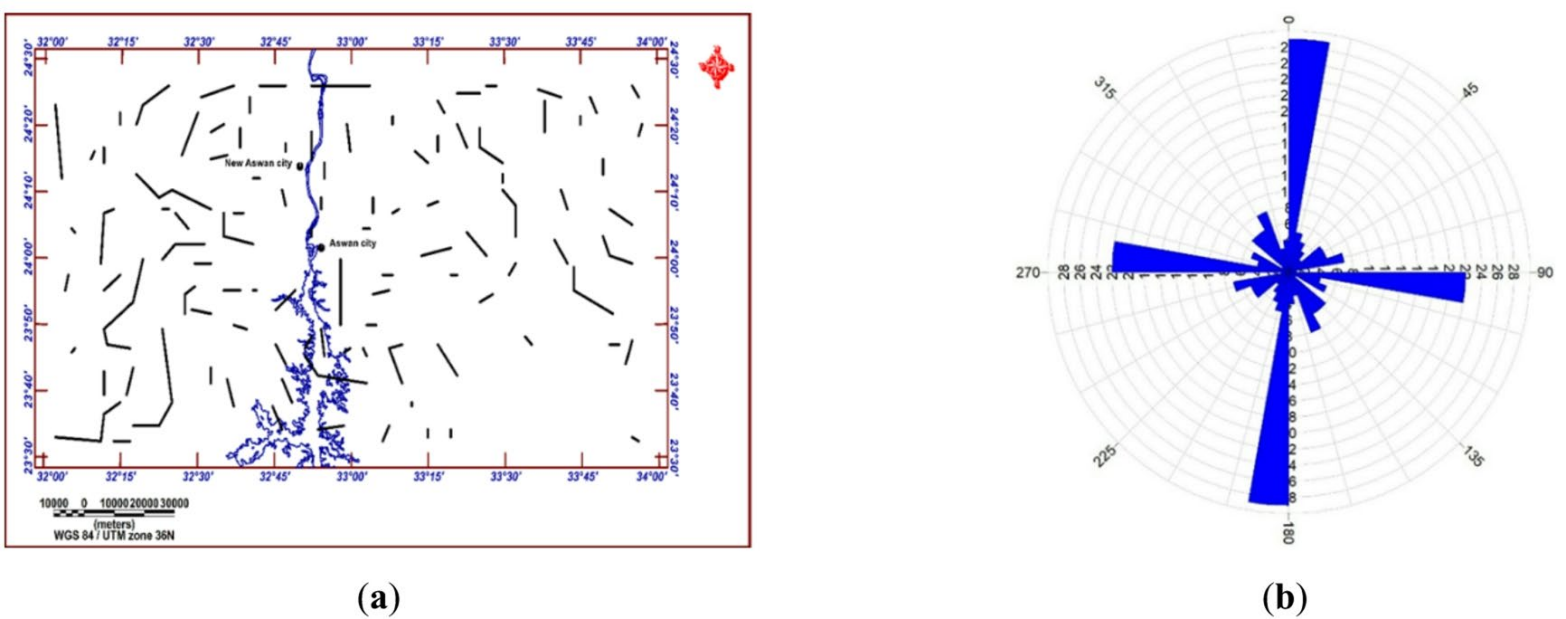
(a) Main structural lineaments deduced from the tilt gravity anomaly map; (b) Their azimuth‑frequency (rose) diagram.

**Fig. 20:**
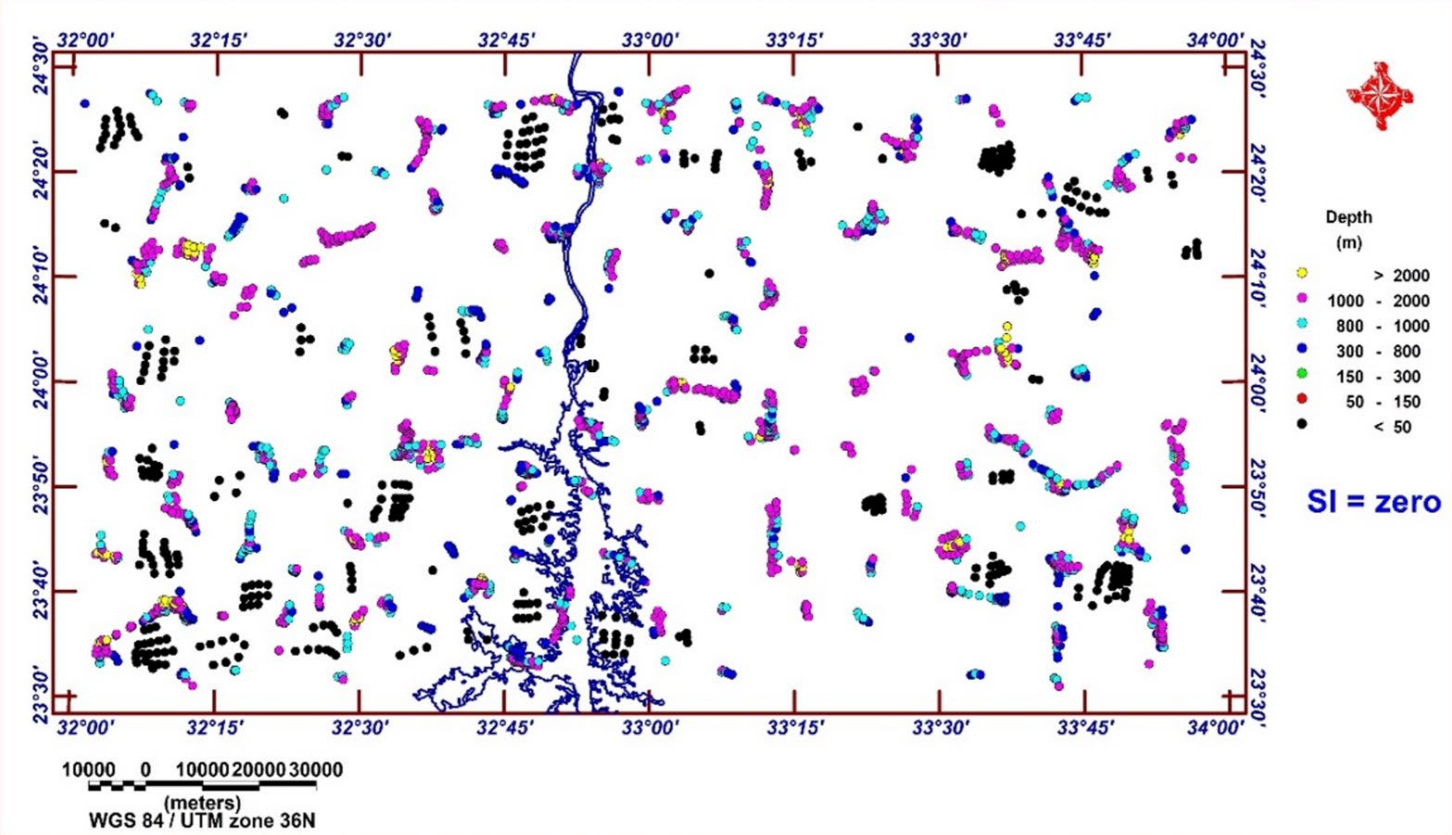
Euler deconvolution applied to the gravity data (FVD) of the study area with a structural index equal to zero.

Wavelength separation using an 8th-order Butterworth filter with a ≈ 200 km cutoff isolates different depth scale contributions in the gravity field and aids interpretation. The low-pass component (Fig. 13) retains very long wavelength structure and highlights regional-scale features likely associated with deep-seated crustal or lithospheric architecture, including broad basin-like depressions and crustal roots. Conversely, the complementary high-pass component (Fig. 14) suppresses the regional trend and enhances intermediate to short-wavelength anomalies that correlate spatially with mapped shallow tectonic elements and putative basement relief; these anomalies are taken as primary indicators of shallower sources relevant to basin geometry and neotectonics faulting. Because the chosen cutoff emphasizes low-intensity signals, interpretations of the structure in the middle or upper crust must be supported by additional filtering tests with smaller cutoffs. Edge enhancement and normalized derivative products further constrain the lateral position of the inductive interface. The first vertical derivative (FVD; Fig. 15) amplifies short-wavelength gradients and produces narrow dipole lobes that facilitate mapping of the horizontal location of density contrasts; Narrow, high-amplitude FVD anomalies are interpreted as originating from shallow sources, while broad derivative signatures indicate deeper causative bodies. The tilt derivative (TDR; Fig. 16) provides a polarity-independent edge-detection metric whose zero-contour closely approximates the projection of density contacts at the surface; TDR therefore serves as a robust guide for tracing continuous fault- and contact-like features across regions of varying anomaly amplitude. Interpretation is performed by integrating derivative products with the raw and filtered Bouguer gravity data to minimize misidentification of noise-amplified artefacts as geological structure. Structural lineament extraction and directional statistics synthesize the spatial pattern of density contrasts and quantify the fabric of deformation. Mapped linear traces^23,24^(Figs. 17, 18 and 19) delineate a system of linear density contrasts interpreted as faults, lithologic contacts, or basement fabrics; corresponding rose diagrams indicate dominant azimuthal trends (for example, N–S and NE–SW orientations) and reveal multiple structural sets, consistent with an overprinting tectonic history. Lineaments that are reproducible across two or more independent gravity products (low-pass, high-pass, FVD, or TDR) are treated as high-confidence features and used to constrain subsequent depth estimation and forward modelling. Euler solutions are fitted to the first vertical gradient of the Bouguer gravity map using (SI = 0). The solutions were plotted as color-coded circles indicating source depths from 5 m to 2000 m (Fig. 20). Euler deconvolution was later used to delineate faults and contact zones at multiple depth levels, which were interpreted as fractures in the basement.

A three-tiered quantitative workflow was applied to convert qualitative map patterns into constrained subsurface models: (1) spectral analysis was used only to determine the appropriate cutoff wavelength for regional–residual separation; (2) Euler deconvolution with structural index (SI = 0) was applied to the first vertical derivative (FVD) of the gravity data and to the RTP aeromagnetic data to estimate the depths and lateral positions of structural contacts and fault-related sources; and (3) targeted 2-D forward modelling along representative profiles was carried out to refine the depth estimates and the physical properties of the causative bodies. To reduce interpretation ambiguity, the derived structural features were systematically compared with DEM-extracted lineaments and remote sensing observations.

### Magnetic Data

**Fig. 21:**
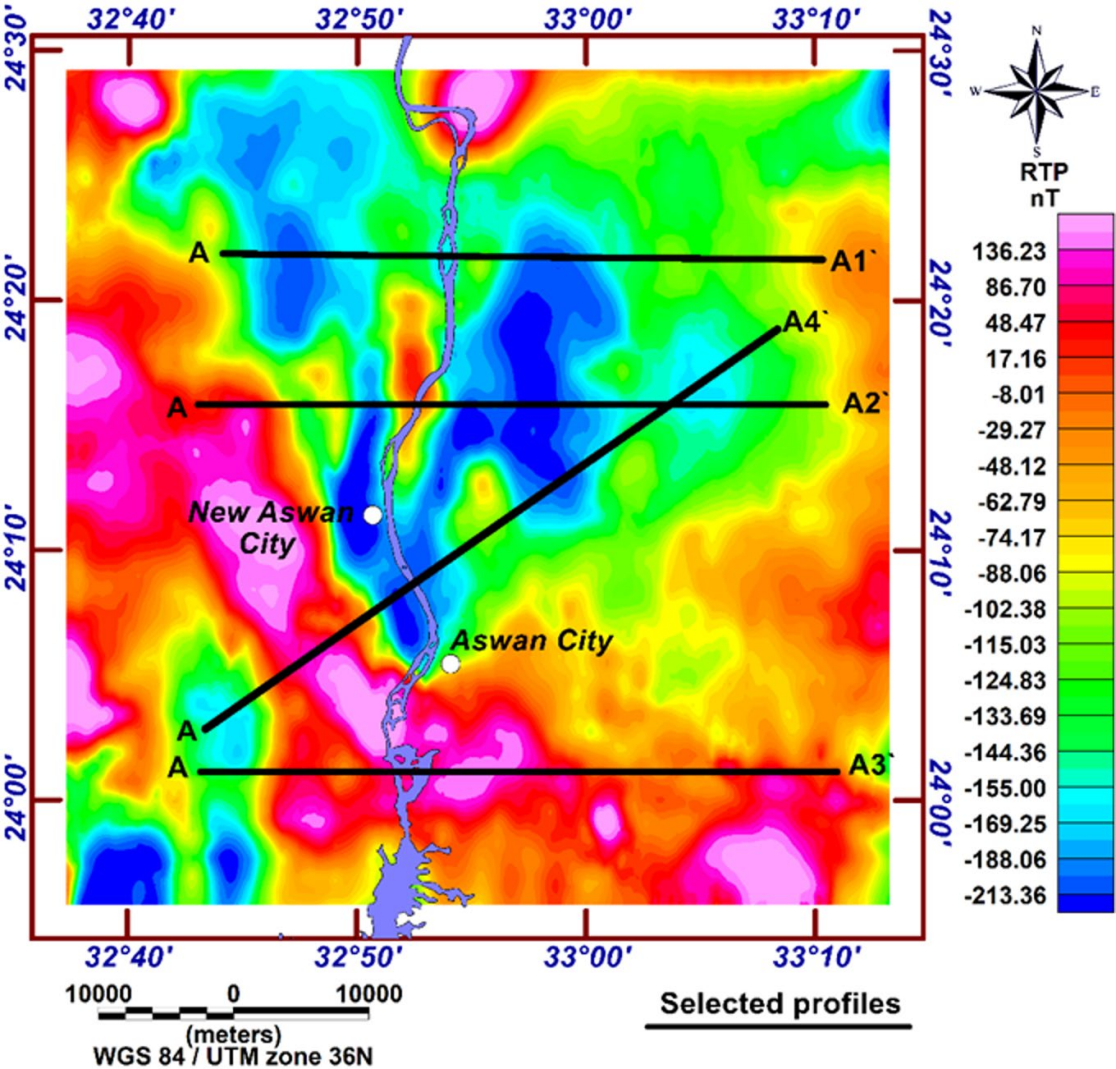
RTP aeromagnetic anomaly map of the studied area with Two-dimensional modeling profiles (A-A1`, A-A2`, A-A3`, and A-A4`).

**Fig. 22:**
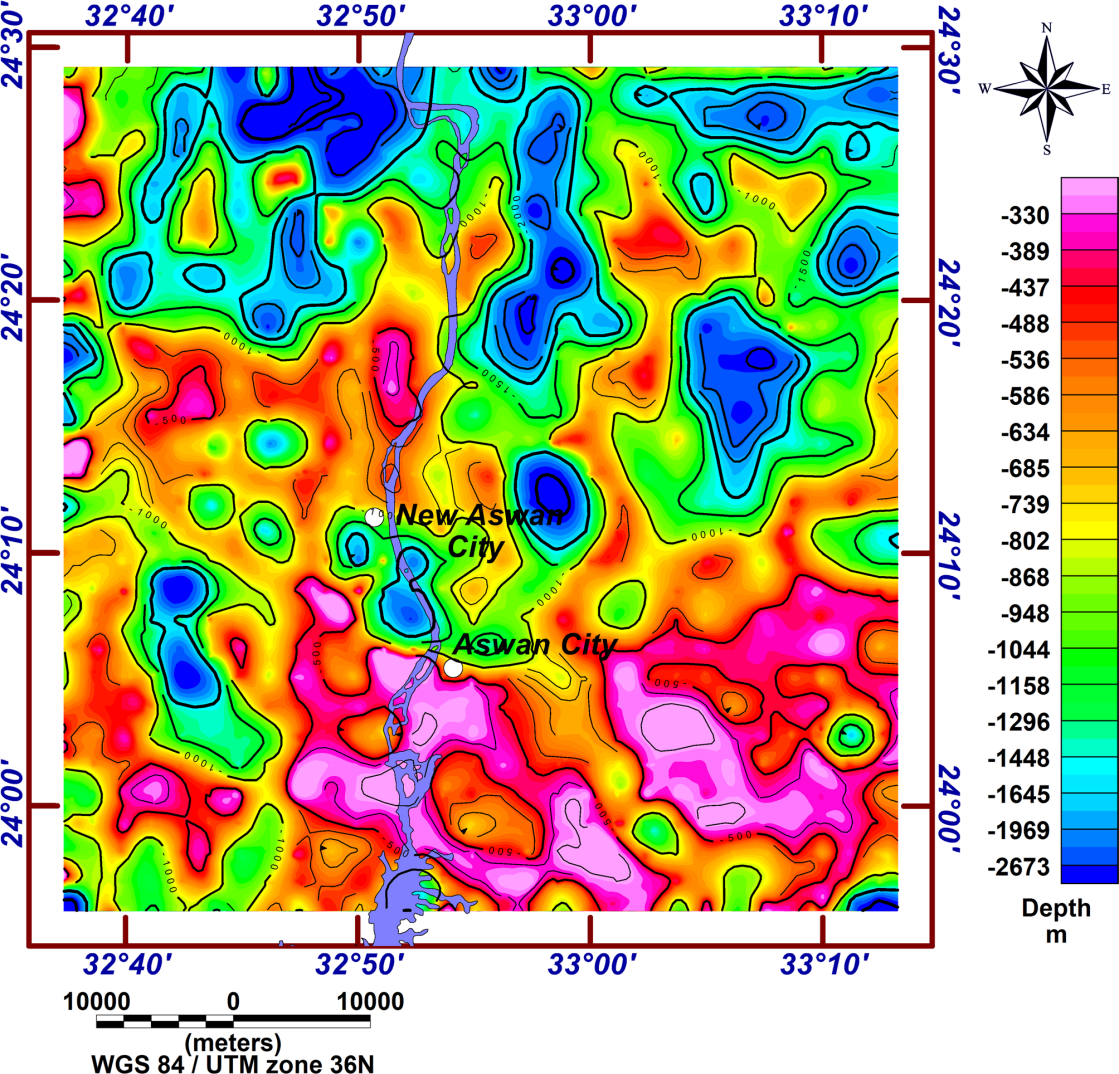
The depths map was obtained by applying the source parameter imaging technique to the RTP aeromagnetic map.

**Fig. 23:**
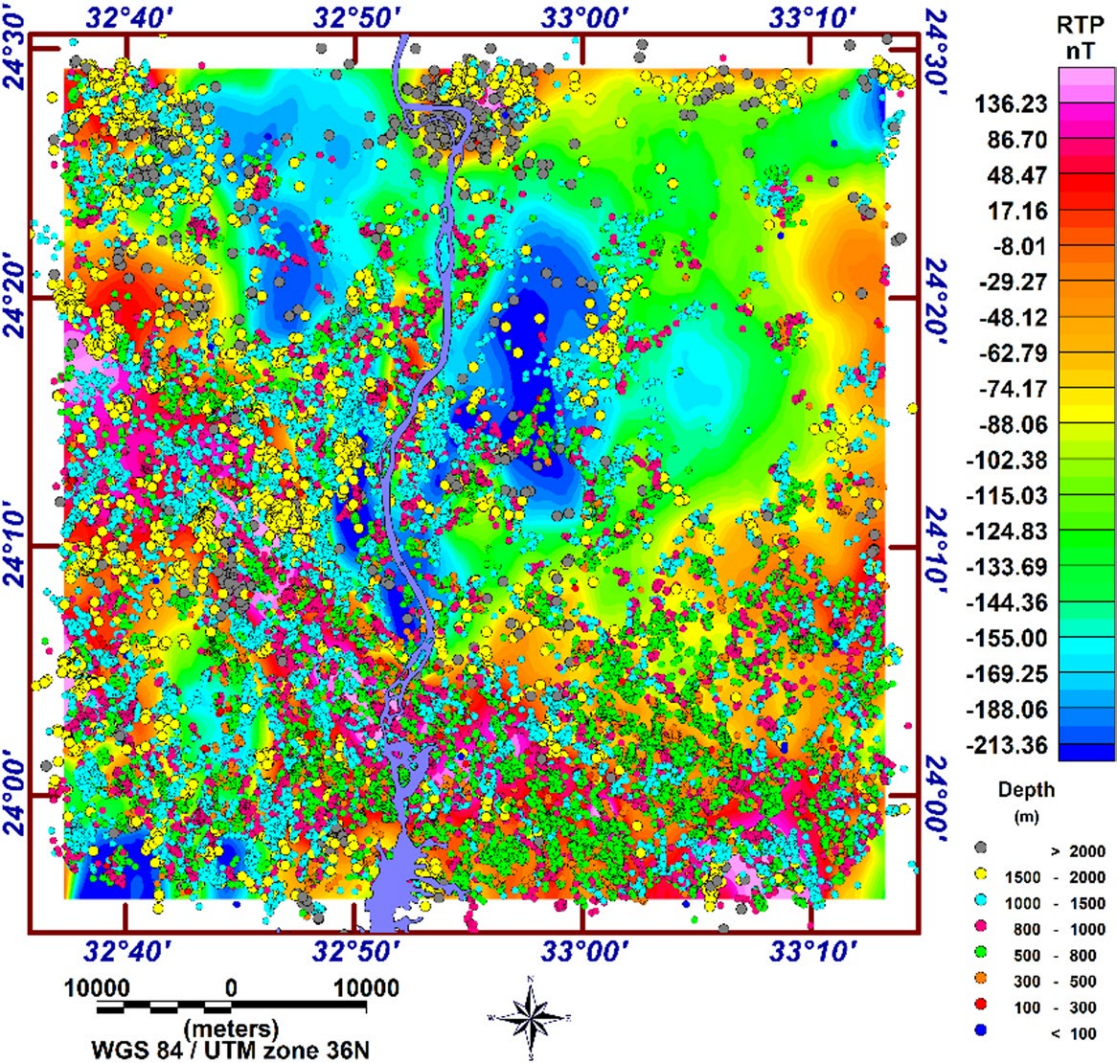
Classified Euler deconvolution solutions derived from the RTP aeromagnetic map of the studied area (structural index (SI) =zero).

The total field magnetic map is reduced to the pole to remove the anomaly effect of the Earth’s main magnetic field using inclination (34.5°) and declination (4.3°E) adopted from the geomagnetic parameters corresponding to the study area and survey epoch, consistent with IGRF values used during data processing. The reduced to pole (RTP) magnetic anomaly map is shown in Fig. 21. Source Parameter Imaging (SPI) was applied to the RTP grid to compute local wavenumber and generate depth-distribution maps (Fig. 22)^25^. Also, three‐dimensional Euler deconvolution (structural index = 0) (Fig. 23) was used to estimate source depths and delineate magnetic contacts^26,27^. Finally, two‐dimensional forward modeling along four selected profiles was carried out in GM‐SYS 2D to iteratively fit observed versus calculated magnetic anomalies (Fig. 21), using a regional field intensity of 41,240 nT, declination 0° E, inclination 90°, and susceptibilities in the range 0–0.004 cgs.

RTP was applied to transform magnetic data so that anomaly peaks were centered over their respective causative sources. This correction enhances the delineation of structural edges and trends and is particularly important for improved reliability of Euler deconvolution results. By better satisfying the assumption that anomalies are located approximately above their sources, RTP facilitates more coherent clustering of Euler solutions along contacts and faults, thereby improving interpretation and depth estimation.

## RESULTS AND DISCUSSION

The fracture network derived from the DEM in the New Aswan–Aswan city block (Fig. 3) shows a dominant primary NW–SE trend, along with its conjugate SE–NW set (Figs. 5 and 6). This orientation is aligned with the sinistral Najd Fault System and the Pan-African shear belts of the Nubian Shield, which corresponds with the late Pan-African convergence event^2,28^. A prominent NE–SW/SW–NE conjugate fracture swarm (Figs. 5 and 6) is associated with R-shear Riedel pairs, formed during the D₃ transgression phase in the Central Eastern Desert^29^. Additionally, a subordinate E–W trend, which is sensitive to binning parameters (Figs. 5 and 6), represents younger extensional structures and unloading joints within the Aswan granite caprock.

The pronounced NW-SE structural grain plays an important role in determining regional drainage patterns, slope stability, and groundwater flow paths in Upper Egypt. Gravity data were useful in characterizing subsurface structural configuration, particularly in areas where surface exposure is limited or buried by sedimentary cover. Gravity data were used to assess the tectonic framework and structural trends controlling the subsurface architecture of the Aswan region and surrounding areas in southern Egypt. The study area is influenced by the Red Sea-Gulf of Suez rift system and is underlain by a complex basement of pan-African origin. Structural framework derived from visual and automated lineament analysis, in combination with geophysical data, guided groundwater and mineral exploration efforts.

Through the integration of these methods, fault and fracture zones controlling subsurface flow pathways for groundwater and mineralization were delineated. In particular, dykes and dyke swarms, which intruded on pre-existing areas of weakness, were highlighted, revealing active faulting patterns beneath their emplacement. Visual assessment of several satellite image products, summarized in Fig. 9, shows that faults play both positive and negative roles in groundwater flow. On the one hand, faults increase permeability by increasing fracture density or by structurally weakening the granitic basement bedrock. On the other hand, faults can impede groundwater flow by creating impermeable fault planes, diverting flow, or forcing water to surface springs^21^.

Conversely, faults commonly impede groundwater flow by producing impermeable fault planes, diverting subsurface movement, or forcing water to emerge at springs^21^. Remote sensing data from Landsat, Sentinel-2, and radar identify N-S, E-W, and NE-SW trending dyke intrusions (Fig. 10) that act as subsurface barriers, although local flow persists where crossed by fracture zones. The dykes are also clearly delineated in the Sentinel-2–derived Modified Normalized Difference Water Index (MNDWI) image (Fig. 11). Moreover, the viewpoint of the Aswan area (Fig. 12) reveals a major fault zone indicated by arrows, demonstrating the effectiveness of lineament analysis in recognizing potential groundwater flow-pathways.

The Bouguer anomaly of the Aswan region (Fig. 7) shows noteworthy lateral variations in subsurface density, with values ranging from − 13.5 mGal (highest) to − 48.3 mGal (lowest). High anomalies, ranging from − 13.5 mGal to − 22.0 mGal, are concentrated around Aswan City and New Aswan City, signifying uplifted and structurally controlled basement blocks and/or denser granodioritic to intermediate intrusive phases within the Precambrian basement. Contrariwise, the lower anomalies, ranging from − 35.5 mGal to − 48.3 mGal, indicated regions of lower-density material, possibly related to deep-seated structural depressions, fractured or altered basement rocks, or localized low-density zones within the Precambrian basement. The abrupt gradients between high/low anomalies point to weighty structural features, such as faults/fracture zones, potentially controlling regional tectonics and influencing the course of the River Nile, which notably follows a structural low in the anomaly field.

To distinguish structural features at different depths, high-pass and low-pass filtering techniques were applied to the gravity data. High-pass filtering (Fig. 14) resolved discrete gravity highs (+ 1.41 mGal) and lows (-1.78 mGal) arranged in linear clusters. These anomalies delineate an N-S-trending lineament parallel to the Nile and an NE-SW-trending lineament to the west, defining a near-surface, tight fault zone. In contrast, low-pass filtering (Fig. 13) emphasized broader regional patterns, highlighting a prominent gravity high (-13.2 to -21.6 mGal) east of New Aswan city and deeper gravity levels (-40.5 to -48.1 mGal) to the northwest and southeast. This regional configuration mirrors a basement surface that exhibits a general structural trend in the NW–SE to E–W directions and has a conjugate system of basement uplift (rift shoulders) and adjacent basin margins.

The structural interpretation was enhanced through application of the first vertical derivative (FVD) and tilt derivative (TDR) techniques. As illustrated in Fig. 15, the FVD map accentuates abrupt variations in density, thereby delineating the margins of intrusive bodies and steeply dipping fault zones. Anomaly values ranging from − 0.002 to 0.001 mGal/m delineate shallow subsurface interfaces, with pronounced positive linear anomalies-oriented north–south between longitudes 32°45′E and 33°15′E (proximal to the Nile), indicative of major fault planes. Additional structural lineaments trending NE–SW and NW–SE converge toward the central sector, whereas intervening negative anomalies correspond to hanging wall blocks or zones of attenuated sedimentary cover.

Tilt-derivative (TDR) maps (Fig. 16) improve subsurface imaging by balancing vertical and horizontal gradient components, thereby resolving density contrasts at different scales. TDR values, which range from − 1.40 to + 1.22, are used to delineate regional fault architecture using zero contour lines. Positive TDR anomalies (+ 0.02 to + 1.22) correspond to the uplifted central block located east of the Nile (32°30′–33°00′E / 23°50′–24°20′N), while negative values (− 0.10 to − 1.40) reflect an adjacent structural depression, which is typically associated with downthrown or subsided zones characterized by lower density contrasts, consistent with basin-like features. The empty contour configuration reveals a multidimensional fault network: dominant N-S trends parallel to the Nile axis, NE-SW-oriented faults west of 32°30′E, and NW-SE connecting structures east of 33°30′E. In the southeastern area, an E-W trending transition bounds a fault bounding a deep sedimentary basin. As a result, the TDR method provides high-resolution identification of fault-controlled fluid migration pathways and potential near-surface resources.

Structural lineaments within the Aswan region were interpreted from Bouguer anomaly, high-pass-filtered, and tilt-derivative maps (Figs. 17 and 18, and 19). To elucidate dominant structural patterns, rose diagrams were generated from the identified lineaments. The prevailing structural orientations are summarized as follows:

- N-S trend (Azimuth 0°/180°): The most prominent orientation in the gravity data, these reflect normal faults that accommodate rift-parallel extension and crustal stretching associated with the Gulf of Suez-Red Sea system^30–32^.
- E-trending (Azimuth 90°/270°): Represents important rift or shear zones, especially between Abu Simbel and Aswan. They connect N-S fault segments and act as primary conduits for fluid migration, controlling groundwater flow and hydrothermal mineralization^31–33^.
- NE-SW trends (Azimuth 45°): Moderately persistent and sparsely represented in the tilt data, these align with oblique Pan-African shear fabrics, suggesting reactivation of ancient basement shear zones^33^.

NW-SE trend (Azimuth 315°): These minor fractures cross-cut dominant N–S faults, interpreted as relict Paleozoic–Mesozoic fractures with no recent activity, suggesting they are dormant lines in a modern rift.

The RTP anomaly map (Fig. 8) exhibits distinct magnetic variations that delineate subsurface structures and regional igneous activity. A prominent anomaly in the southwestern part, reaching approximately 42,580 nT, demarcates a major NW–SE-trending fault zone. This structural feature is interpreted to represent a tectonic boundary separating the eastern and western structural domains. The low magnetic anomalies observed in the northern, central, and northwestern parts are interpreted to reflect zones of relatively lower magnetic susceptibility and enhanced structural disruption within the basement rocks, whereas the high anomalies correspond to more coherent and magnetically stronger basement domains associated with variations in lithology and structural configuration.

In this study, a structural index (SI) of 0 was utilized for Euler deconvolution of gravity and magnetic data to assess geological features characterized by contacts, faults, and abrupt lithological transitions. This choice aligns with the predominant structural framework of the Aswan region. While other indices, such as SI = 2 for cylindrical formations or SI = 1 for dyke-like structures, could better describe specific intrusive geometries, the analysis primarily emphasizes regional-scale structural trends and major tectonic discontinuities rather than the detailed classification of source types. Consequently, selecting SI = 0 provides a stable and uniform representation of the overarching structural framework. The Euler solutions obtained were further corroborated by integrating them with interpretations of gravity and magnetic anomalies, as well as results from 2D forward modeling.

Source Parameter Imaging (SPI) analysis alongside Euler deconvolution results (Figs. 21 and 22, respectively) offers complementary insights into subsurface source depths and structural boundaries. According to SPI analysis, subsurface depth varies considerably across the study area. The shallowest sources, ranging from approximately 350–400 m deep, are located in the southern, southwestern, and southeastern areas, whereas the deepest sources, reaching depths between 1,800 and 2,600 m, are found in the northern, north-central, and northeastern regions. Similarly, Euler deconvolution results based on an SI value of 0 reveal linear clusters with a dominant NW–SE orientation, which are interpreted as contact zones and basin–basement boundaries. The depth estimates derived from Euler analysis range from approximately 1,500 to 2,500 m, aligning closely with the deeper levels identified through SPI. This convergence between SPI results and Euler solutions bolsters confidence in the reliability of the interpreted structural framework and substantiates the presence of significant fault-controlled basement variations throughout the study area.

**Fig. 24:**
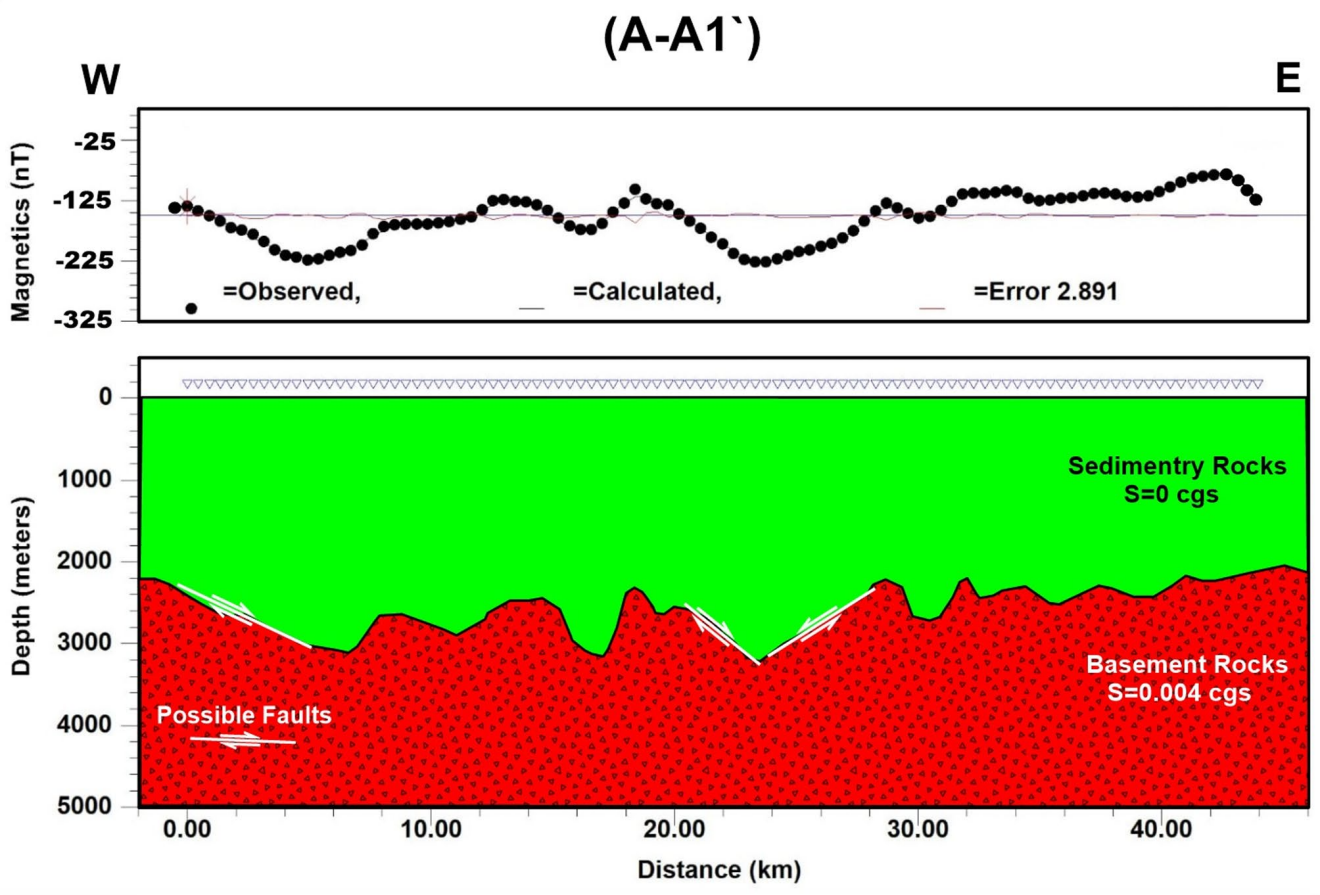
Two-dimensional model along profile (A-A1`) of the RTP aeromagnetic map.

**Fig. 25:**
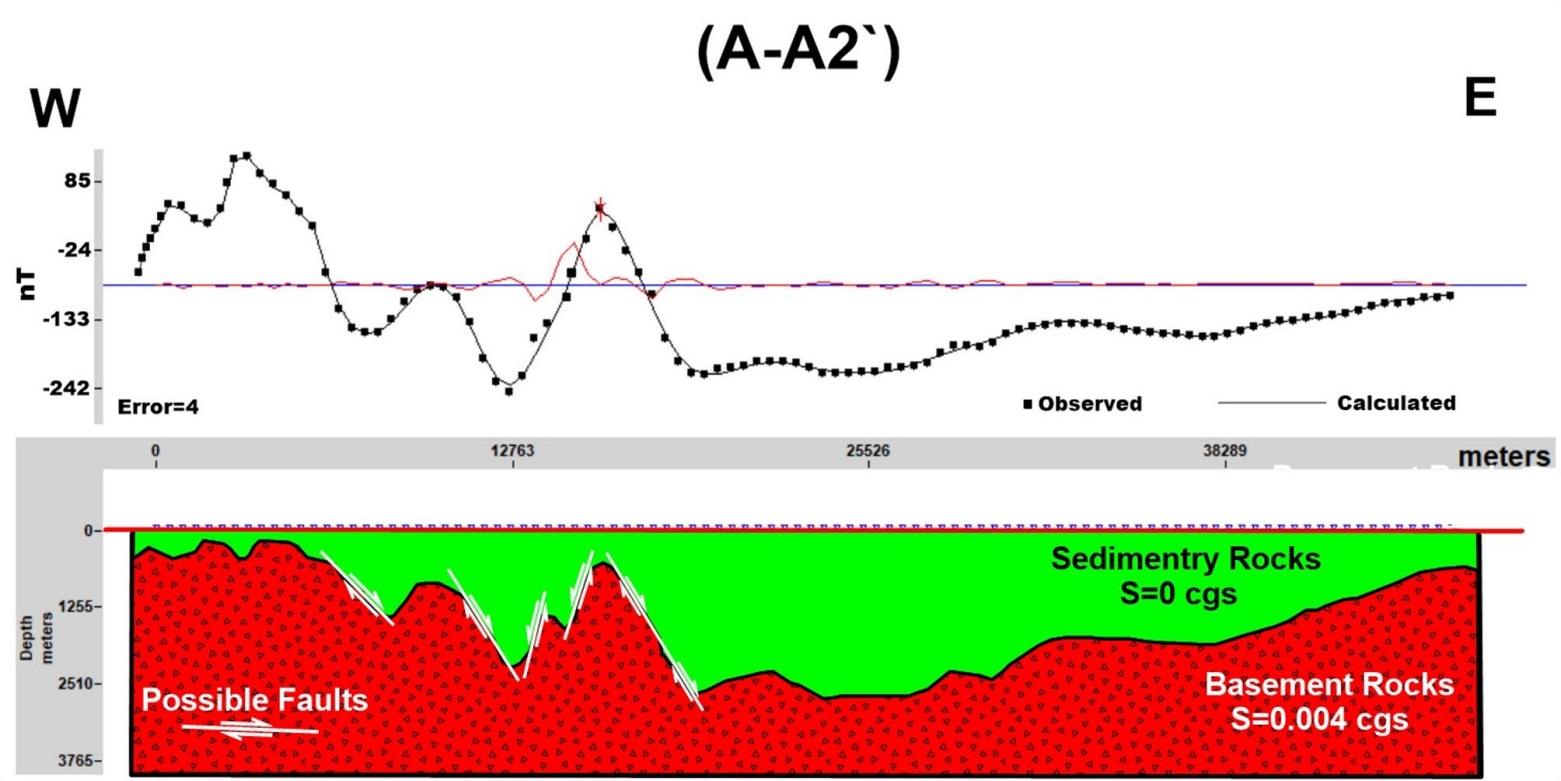
Two-dimensional model along profile (A-A2`) of the RTP aeromagnetic map.

**Fig. 26:**
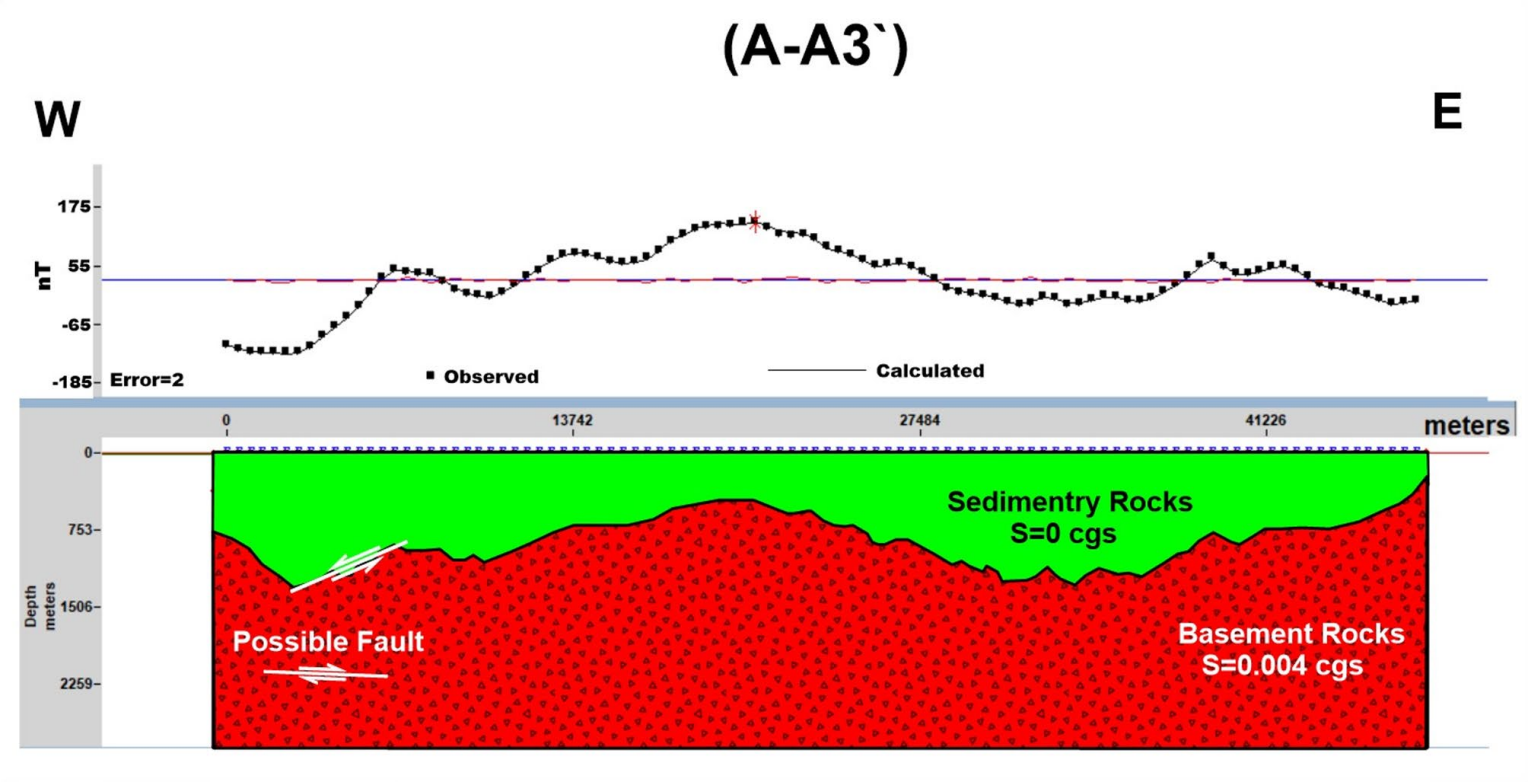
Two-dimensional model along profile (A-A3`) of the RTP aeromagnetic map.

**Fig. 27:**
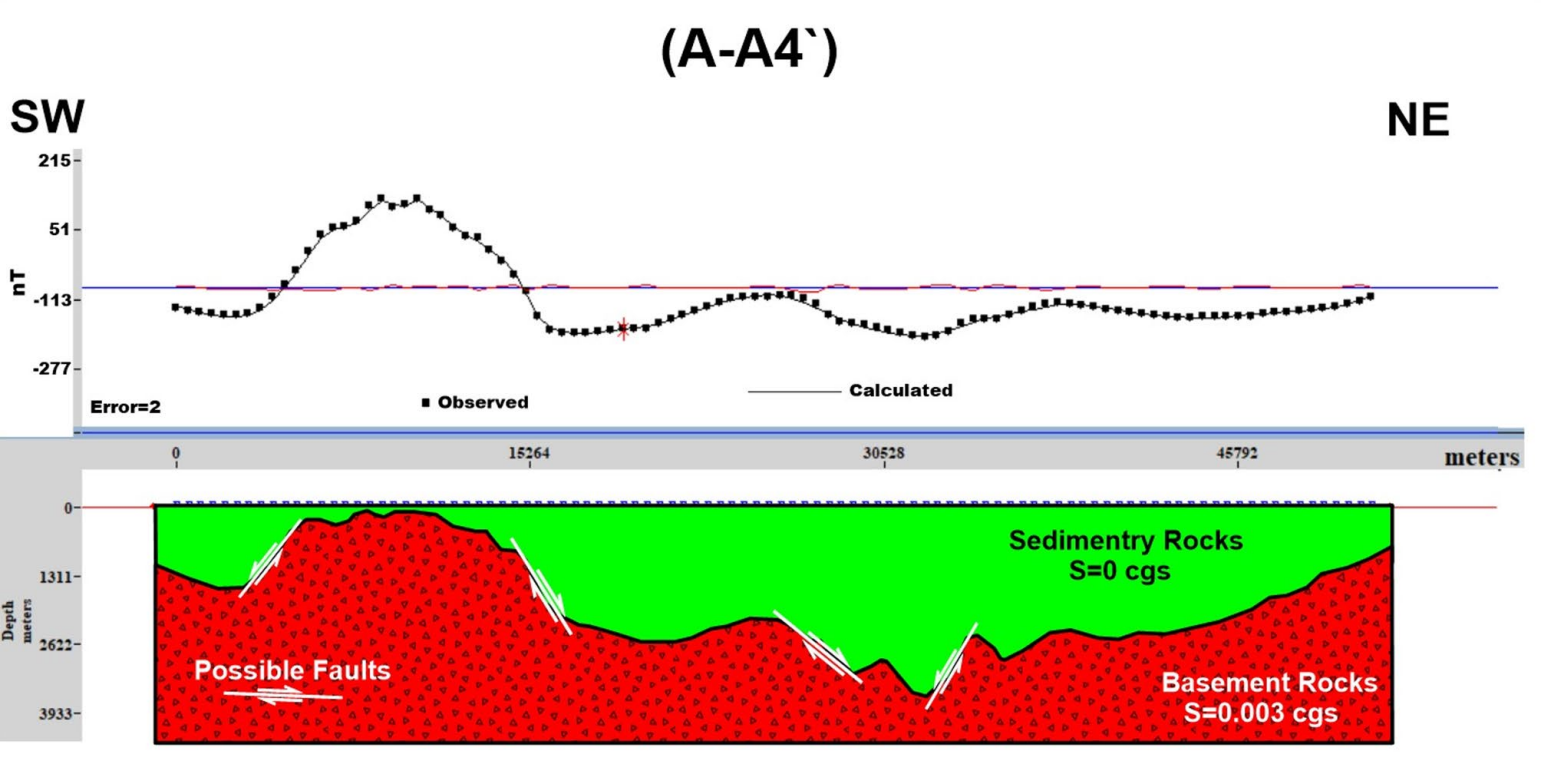
Two-dimensional model along profile (A-A4`) of the RTP aeromagnetic map.

Forward 2D magnetic modeling (profiles A-A1′ to A-A4′; Figs. 24, 25, 26 and 27), controlled by SPI and Euler projections and Bouguer trends, reproduces the observed RTP anomalies and constrains the basement geometry at depth. Model results indicate basement tops with varying profiles from 0.35 km to 3.0 km, with shallow basements (0.35–1.10 km) in the uplifted blocks and deep depocenters (2.3–3.0 km) in the central troughs. The sensitivity of the modeled basement is 0.002–0.004 cgs, with the upper sediments in the model being effectively non-magnetic.

The integration of DEM, gravity, and magnetic datasets generates a self-contained, multiscale structural model. Surface lines derived from DEM and imagery coincide with shallow gravity edges (high pass, FVD), which define N-S, NE-SW, and NW-SE network structures that control drainage, slope instability, and near-surface permeability. Regional gravity signatures (Bouguer high/low and tilt/FVD maps) identify basement uplift and basin margins corresponding to deep magnetic highs and SPI/Euler depth patterns. Magnetic depth solutions and 2-D forward models measure variations from depth to basement (0.35–3.0 km) and validate the location of basin depositors inferred from gravity. Dyke swarms mapped in remote sensing products are spatially correlated with distinct magnetic and gravity anomalies; these intrusions locally modulate permeability, acting as barriers when intact or as conduits when weathered, thereby contributing to hydraulic compartmentalization.

A key factor in interpreting depth estimates obtained from SPI, Euler deconvolution, and 2D forward modeling is the uncertainty linked to the computed values. In this study, the calculated depths are thus shown as estimated ranges instead of fixed, certain values. The reliability in depth estimation is assessed through the agreement of independent geophysical techniques and the stability of results across profiles. As a result, the total uncertainty is projected to be around ± 10–15%, indicating fluctuations due to method sensitivity, data resolution, and model assumptions. This multi-faceted agreement boosts trust in the dependability of the interpreted structural depths.

## CONCLUSIONS

The integrated analysis of DEM-derived lineaments and gravity data offers a comprehensive understanding of the structural framework in the Aswan region. The lineaments identified through DEM analysis predominantly exhibit a NW–SE trending Pan-African shear fabric, accompanied by secondary E–W structural trends that are interpreted as joint or fracture systems. This configuration highlights the reactivation and exhumation of ancient basement structures, shaped by regional tectonic stress release.

Gravity-based analyses, including Bouguer anomaly mapping, high-pass filtering, and tilt-derivative techniques, reveal prominent N–S trending deep-seated structures. These features are interpreted as rift-parallel normal faults associated with the extensional regime of the Gulf of Suez–Red Sea system. The identified trends align with observations from surface structural analyses, indicating a strong continuity of structures at depth. Furthermore, both gravity and DEM datasets detect NE–SW trending features interpreted as reactivated Pan-African shear zones, influenced by current stress fields. Gravity data uniquely highlight E–W trending transfer zones that connect N–S fault segments, potentially serving as conduits for deep fluid migration. However, these features are less visible in surface topography, likely obscured by geomorphic processes.

The integration of RTP aeromagnetic and Bouguer gravity data effectively reveals the subsurface structural configuration and basement topography of the Aswan region. Depth estimates derived from techniques such as Source Parameter Imaging (SPI), Euler deconvolution, and 2D forward modeling indicate significant variation in basement depths, ranging from approximately 300 m to 2,300 m across the study area. These findings demonstrate a structurally complex subsurface architecture, which is crucial for understanding the region’s tectonic evolution. Additionally, the results provide valuable insights for applications such as groundwater resource evaluation, seismic hazard assessment, and further geophysical explorations.

## Data Availability

The data that support the findings of this study are available from the corresponding author upon reasonable request.
